# Tuning Pore Confinement and Chemistry of Fluorine-Free Membranes

**DOI:** 10.1021/jacs.6c15621

**Published:** 2026-10-09

**Authors:** Muhammad Saad Naeem, Zan Lian, Jordi Guardià, Ranga Rohit Seemakurthi, Pol Sanz-Berman, Marta Giamberini, Ricard Garcia-Valls, Núria López

**Affiliations:** † 202569 Institute of Chemical Research of Catalonia (ICIQ-CERCA), The Barcelona Institute of Science and Technology, (BIST), Av. Països Catalans 16, Tarragona 43007, Spain; ‡ 16777 Universitat Rovira i Virgili, Campus Sescelades, N4 Block, C. Marcel·lí Domingo 1, Tarragona 43007, Spain; § Department of Analytical Chemistry and Organic Chemistry, Universitat Rovira i Virgili, CarrerMarcel.lí Domingo 1, Tarragona 43007, Spain; ∥ Department of Chemical Engineering, Universitat Rovira i Virgili, Av. Països Catalans, Tarragona 26 43007, Spain

## Abstract

The design of low-temperature fuel cells and electrolyzers involving proton exchange membranes (PEMs) has been dominated by the exceptional performance and industrial dominance of Nafion. With emerging regulations banning the use of fluorinated compounds, the search for F-free alternatives must be accelerated. Here, we optimize a fluorine-free, sustainable, and low-cost liquid crystalline poly­(epichlorohydrin)­membrane, based on the identification of key descriptors for proton mobility obtained using machine learning interatomic potentials (MLIPs) derived from first-principles calculations for extensive molecular dynamics. Proton transfer is controlled by the interplay between water confinement and internal pore chemistry. This allows us to modify the active functional groups so that they directly participate in the proton diffusion process, increasing three times the diffusion rate in the membrane, thus being closer to Nafion standards. This work provides key descriptors for a targeted design approach essential for catalyzing the commercial development of next-generation fluorine-free PEMs.

## Graphical abstract

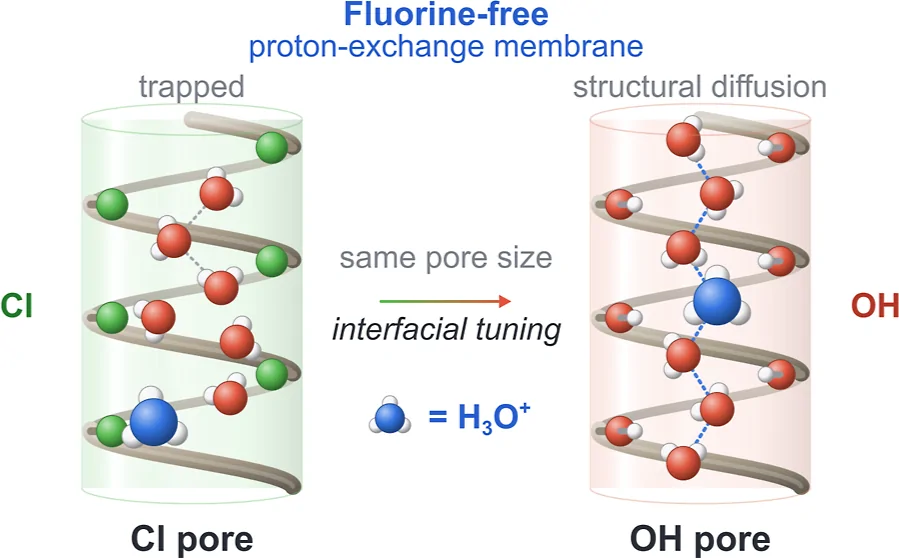

## Introduction

Electrolyzers and fuel cells are fundamental in shaping a decarbonized future.^1,2^ These electrochemical devices have a key component, the membrane that separates the anolyte and catholyte compartments while facilitating effective ionic selectivity and transport. For electrolyzers, the robust industrial technologies rely on alkaline exchange membranes, although they present moderate efficiencies. Higher efficiencies can be obtained with proton exchange membranes (PEMs); other solutions based on anion exchange or bipolar membranes are precommercial.^3–7^

As for PEMs, membranes are based on perfluorosulfonic acid (PFSA), with the Nafion series from DuPont remaining the prevalent commercial choice since their introduction in the 1970s.^8,9^ Nafion consists of a phase-separated structure composed of a hydrophobic polytetrafluoroethylene (PTFE) backbone and hydrophilic sulfonic acid side chains responsible for its functionality.^9–13^ As a highly optimized material, Nafion presents unique proton conductivity, electrochemical stability, and mechanical endurance under relevant operating conditions.^5,14–16^ Notably, Savage et al.^17,18^ utilized explicit proton transport simulations to demonstrate the key interplay between water structures and the flexibility of sulfonate side chains, identifying a “proton swapping” mechanism between sulfonate groups that is fundamentally dictated by the level of hydration. This understanding is explored by numerous studies inspecting structural features,^19,20^ properties,^9^ proton transport mechanisms,^21–24^ water dynamics under confinement,^25–28^ and degradation.^29,30^

Despite these excellent properties, Nafion suffers under intense operating conditions with challenges such as proton conduction trade-off under humidity, mechanical degradation under high water content, and thermal instability under high temperatures (>200 °C).^9^ Moreover, the ban on all per- and polyfluoroalkyl substances (PFAS)—commonly referred to as “forever chemicals”—proposed by the European Chemicals Agency^31,32^ might pose an end to Nafion supremacy. Therefore, the accelerated search for nonfluorinated alternatives,^33–35^ delayed for many years, is now urgently needed.

The key property of membranes is the selective proton transport. In bulk water, the fast proton diffusion (*D*_H_+ = 9.5 × 10^–5^ cm^2^/s, experimental value at 30 °C) has been proposed in a recent machine learning neural-network-based molecular dynamics study to be gated by two sequential hydrogen-bond exchanges where the identified barriers are on the order of *k*_*B*_ *T*.^36^ However, ab initio molecular dynamics (AIMD) simulations have shown that multihop events are the result of proton rattling and that the “special-pair dance” remains an alternative description of the process.^37^ The Eigen (H_9_O_4_^+^)^38^ and Zundel­(H_5_O_2_^+^)^39^ cations are idealized limiting structures of this so-called Grotthuss mechanism rather than discrete, fully formed species.^40^ A configuration is Zundel-like when the transferring proton sits equidistant between the donor and acceptor oxygens along the transfer coordinate and Eigen-like when it is fully committed to one oxygen. Recent simulations suggest that the hydrated proton is most likely described as a “distorted Eigen” cation, which dynamically switches its “special-pair” partner among neighboring water molecules in a process known as the “special-pair dance”.^41–43^ Complementing this hopping process, vehicular diffusion, i.e., protonated water clusters such as H_3_O^+^, can also physically transport water through the medium. Unlike bulk water, proton transport in nanoconfined membrane environments is governed by complex hydrogen bonding networks resulting from geometric constraints and water–membrane interactions.^44–49^ Ionic diffusion in the membranes, particularly Nafion, is modulated by the degree of hydration, the functional groups of the host, and the diameter of the pores.^50–53^ For Nafion, the high water uptake of around 30% modifies the mesostructure^10–12^ and thus pore size. This fact, together with the number of –SO_3_H acidic sites in the polymer, results in a complex interplay between the Grotthuss mechanism and vehicular diffusion. This leads to a wide range of diffusion values from 10^–6^ to 2 × 10^–5^ cm^2^/s, strongly depending on the hydration level.^54,55^

The search for highly conductive proton membranes has been limited by enormous challenges like high costs, operational stability, and scalability issues. But also more fundamental issues, like the difficulty to accurately model the disordered nature of the polymers and the coexistence of different mesostructures as a function of the water uptake, have limited the opportunities for rational design. Alternative materials such as fully hydrocarbon-based^56,57^ and composite membranes^58,59^ are being investigated. Particularly, fluorine-free self-organized polymers with localized cylindrical ionic channels composed of a conductive hydrophilic backbone and dendritic side groups have gained significant attention.^60–67^ The chemical tunability of these systems, coupled with their skeletal protection against the harsh chemical environment, relatively high ordering, and the channel allowing humidity-independent localized proton transport, are appealing properties. Giamberini et al.^68^ developed liquid crystalline polyethers (SCLCPs) composed of poly­(epichlorohydrin) (PECH) and poly­(epichlorohydrin-*co*-ethylene oxide) (P­(ECH-*co*-EO)) dendronized with potassium 3,4,5-tris­[4-(*n*-dodecan-1-yloxy)­benzyloxy]­benzoate side groups.^69,70^ Combining their columnar structure and geometrical tunability, these liquid crystalline membranes hold significant promise for addressing current challenges. Moreover, the semicrystallinity of PECH enables the atomistic-level details that govern proton transfer through first-principles molecular dynamics. Integrating these experimental insights with theoretical modeling will elucidate the complex dynamic nature of the membrane and accelerate membrane development for technical implementation.

In this study, we demonstrate that the tubular nature of the fluorine-free liquid-crystalline PECH-based membrane and the inner functional groups are crucial for proton diffusion. We obtain two descriptors, the pore size and basicity of the functional groups, as key in proton transport enhancement. We then engineer a modified version of the membrane that can be low-cost and sustainably synthesized from glycerol and enhance proton diffusion, demonstrating the viability of this approach for next-generation fluorine-free PEMs.

## Results and Discussion

### Model Description and Construction

We report experimental synthesis and characterization of the PECH membrane to establish its columnar semicrystalline architecture and the structural characteristics essential for modeling the system. The assumptions underlying the modeling approach are described and validated with the key experimental fingerprints.

Poly­(epichlorohydrin) (PECH) is a flexible polyether with chloromethyl side groups. When attached to dendritic side groups, it can self-assemble into columnar structures.^68^ In the initial study,^68^ PECH was modified by reacting it with potassium-3,4,5-tris­[4-(*n*-dodecan-1-yloxy)­benzyloxy]­benzoate­(TAP) and tetrabutylammonium bromide (TBAB) (at 65 °C for 8 days). The membranes were prepared by the immersion precipitation process, and the columns were oriented using a thermal treatment (heating the membrane above the copolymer clearing temperature followed by slow cooling to the liquid crystalline phase). For the detailed synthesis, characterization, thermal treatment, and resulting PECH modification degrees and yields, see Supporting Information (SI): Experimental section and Table S1. The modification degree characterized by ^1^H NMR (Figure S1) and elemental analysis reaches a plateau at 75% due to steric hindrance from the bulky TAP groups and conformational changes in the polymer, making PECH75 (i.e., 75% PECH units side-modified) the closest to fully modified PECH. Based on the lab-scale pricing including characterization and yields, this membrane is estimated at 2–3 times the current cost of Nafion 117; see the SI section: Cost Estimation.

The XRD for the PECH75 membrane showed *d*-spacing reflections in the ratio of 1:1/ $\sqrt{3}$ :1/2 and a diffuse halo at the high-angle region (2θ = 20°), confirming the presence of a hexagonal columnar mesophase (Col_h_). The first three reflections correspond to the planes (100), (110), and (200), respectively, while the broad halo is related to (001) (Figure S2a). The hexagonal columnar LCP structure can be homeotropically oriented through the thermal treatment, as shown by the XRD azimuthal scan and the Debye pattern (Figure S2b). The HR-TEM image of the PECH75-oriented membrane shows contrast between the aromatic and aliphatic regions of the tapered side groups, clearly demonstrating the presence of homogeneously oriented columns (Figure S3).

The membrane is a columnar liquid crystal polymer composed of a hydrophilic PECH chain attached to the hydrophobic TAP side group (Figure 1). The hydrophobic groups drive the polymer self-assembly and orient outward from the pore center, inducing a helical conformation to the hydrophilic PECH chain in the *z*-axis^68^ (Figure 1a). As a result, the membrane functions as a channel, where proton transport is localized in the helical PECH chain protected by the side hydrophobic groups.

**Figure 1:**
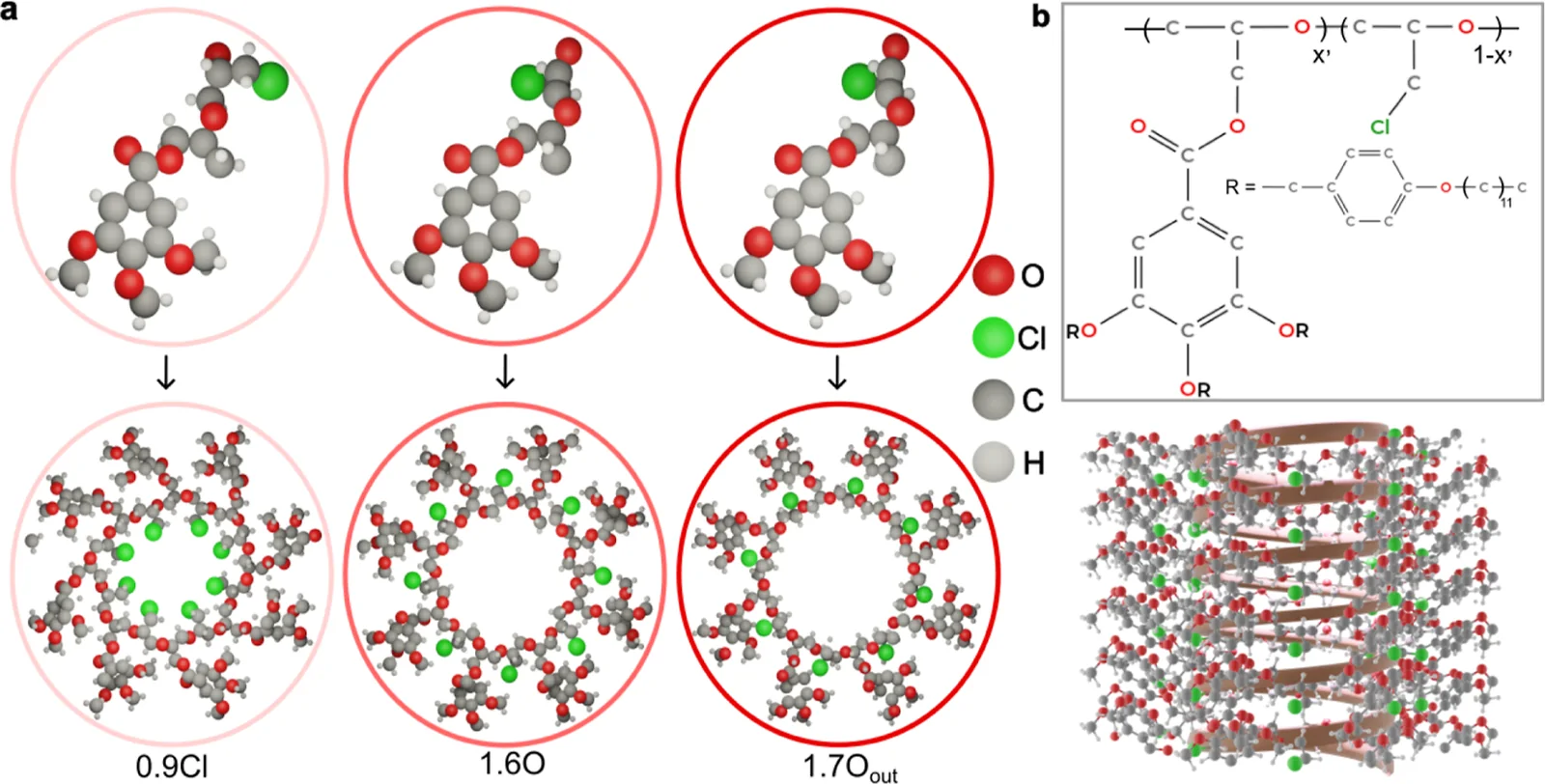
(a) Optimized monomers and corresponding membrane models 0.9Cl, 1.6O, and 1.7O_out_ with their respective pore sizes. (b) Poly­(epichlorohydrin) (PECH); R = potassium 3,4,5-tris­[4-(*n*-dodecan-1-yloxy)­benzyloxy]­benzoate. The pore size variance due to the chloromethyl rotation and the C–O–C bond angle also results in distinct pore environments in each model. The Cl resides inside the 0.9Cl pore, while 1.6O and 1.7O_out_ differ in the inward and outward orientations of ether oxygens.

As the chain orientation and helical conformation are key to the membrane structure and function, we modeled the potential conformers of a single PECH unit attached to a shortened hydrophobic group at the DFT-PBE-D3 level of theory, shown in Figure 1—hereby referred to as a monomer. The lateral chains are capped with H atoms instead of the residual, R, groups to reduce computational expense while keeping mechanical constraints. Three monomer conformers were optimized based on the chloromethyl rotation and C–O–C bond angle (Figure 1a; top row). Next, the monomer is used to model the membrane in two steps: the monomer is repeated to form a ring-shaped helical chain, and the optimized chain is then repeated in the *z*-axis to create the vertical column. An individual helical chain ring, shown from the top in Figure 1a (bottom row), is a chain of eight repeating monomer units, each slightly tilted in the *z*-direction to induce helicity. The tilted distance was kept constant for each monomer to achieve a total ring thickness close to the experimental XRD value of 4.5 Å. After vertically repeating the optimized rings, one of the membrane models is shown from the side in Figure 1a (bottom row).

The procedure is repeated for all three conformers, resulting in three membranes with a single vertical column where the pore size resulting from varying internal functional groups is 0.93 nm, 1.63 nm, and 1.77 nm, and the inter-ring spacing in all the optimized models is ≈ 4.1 Å (Table S2). For further model details and experimental validation, see SI: Model Construction. Thus, the optimized models differ in the pore size and chemical environments inside the pore; the nomenclature stands for the pore size (nm) and the inner functional group. In 0.9Cl, the chlorine atoms reside inside the pore, while the ether oxygens either orient toward or away from the pore center in 1.6O and 1.7O_out_, respectively.

To run the extended molecular dynamics required for the water-structure and transport analyses that follow, we trained a multihead MACE (version 0.3.15)^71^ machine learning interatomic potential (MLIP) with a shared representation and three functional-specific heads (PBE-D3, revPBE-D3, and r2SCAN) on 11,332 reference configurations in total (4,597 PBE-D3, 3,418 revPBE-D3, and 3,317 r2SCAN). We report the production of MACE molecular dynamics at the revPBE-D3 level, as it reproduces the experimental bulk-water structure and removes the overstructuring of PBE-D3 water^72^ (Figures S4, S5, and S6). The corresponding training and validation protocols including water structuring are detailed in the SI section: MACE.

### Confined Water Structures

Experimentally, the water uptake of PECH75 membranes was measured by immersing dry membranes in deionized water at room temperature for 72 h. The membranes were weighed both before and after immersion, resulting in a water uptake of 7.5 ± 0.5 wt % for unoriented PECH75, while the oriented PECH75 shows 3.3 ± 0.5 wt %, see Table S3.

Therefore, the three membrane models were solvated with water molecules, see SI: Pore hydration. In short, water molecules were added sequentially into the pore until the differential energy per water molecule exceeded that of the water dimer (around –0.2 eV). The numbers of water molecules to fill the ring required for 0.9Cl, 1.6O, and 1.7O_out_ are 13, 31, and 32, respectively. The resulting theoretical water uptake is close to the experimental value for unoriented PECH75 (7.5% vs 9.0% for 1.6O, Table S3). As we model a single column, the unoriented system provides an appropriate comparison.

On these fully water-filled systems and bulk water, we run molecular dynamics using the multihead MACE (revPBE-D3, 1 ns, 300 K, NVT, at 0.5 fs time step) to characterize the water structure (Figure 2). The O–O radial distribution function (RDF) shows that the first-neighbor O–O peak stays at 2.8 Å in every pore, matching bulk water, while the second solvation shell (4.5 Å) disappears in all membranes (Figure 2a). The first-shell water coordination is highest in the 1.6O pore (7.4 water neighbors within 3.5 Å) despite being more confined than 1.7O_out_ and lowest in 0.9Cl (5.2). The RDF between water hydrogens (H_w_) and each pore’s wall group probes hydrogen bonding between the water and the pore surface (Figure 2b). In the ether-lined 1.6O and 1.7O_out_ pores, the water hydrogens donate a directed hydrogen bond to the ether oxygen (O*), giving a sharp first peak at 1.8 Å. In 0.9Cl, no such bond forms, as evidenced by a broad peak near 3.1 Å.

**Figure 2:**
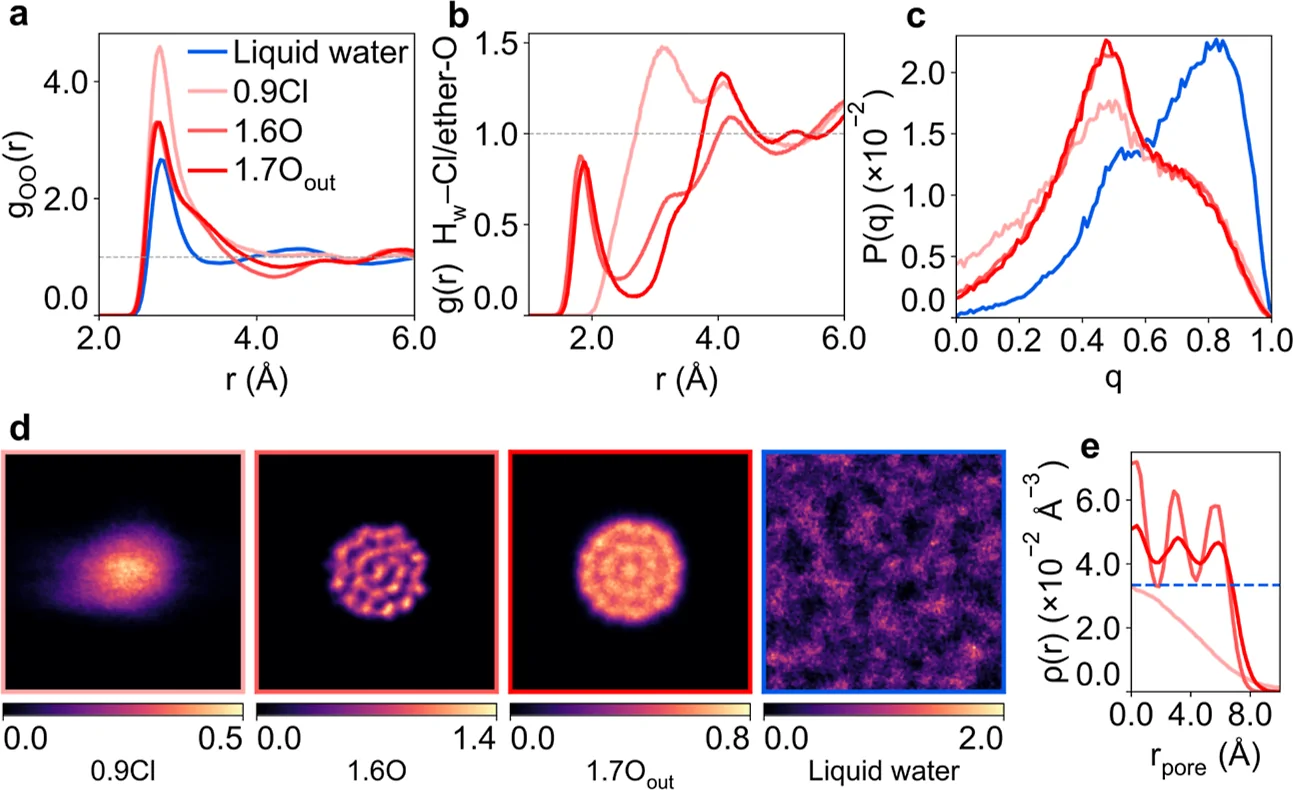
Structural characterization of confined water in the membranes compared with bulk liquid water, computed with the revPBE-D3 head of the MACE model. (a) Water O–O radial distribution function; (b) hydrogen-bond RDF between water hydrogens (H_w_) and each pore’s wall group (Cl for 0.9Cl; ether O for 1.6O and 1.7O_out_); (c) orientational tetrahedral order *q*; (d) 2D density distribution of water oxygens in the pore cross-section, shown without the membrane for clarity; (e) radial density profile ρ­(*r*) measured from the pore axis. The first-neighbor O–O distance (2.8 Å) is preserved under confinement, but the tetrahedral order *q* falls below the bulk value in every pore and is lowest in the subnanometer 0.9Cl pore. The 2D density distribution function shows highly disordered water oxygens for 0.9Cl compared to an ordered ring-like structure in 1.6O and 1.7O_out_.

The orientational tetrahedral order parameter *q* measures the angular order of the four nearest water oxygens and shows 0 for an ideal gas to 1 for a regular tetrahedron.^73,74^ Confinement lowers *q* below the bulk-water value (0.70) in every pore (Figure 2c, Table S2). The disorder is largest in the subnanometer 0.9Cl pore (*q* = 0.37). The same disorder shows in the O–O–O angle distribution, which broadens away from the tetrahedral 109.5° in every pore and most in 0.9Cl, where the water oxygens sit at 3.3 Å from the chloride (Figure S7).

The water structure is visualized through the 2D density distribution of water oxygens for bulk and membrane-confined water (Figure 2d). The liquid water shows broadly distributed water oxygens compared to the closed-packed water structure in the confined systems. Water oxygen atoms in 1.6O also form distinct, concentrated clusters along the concentric rings, indicating water–ether oxygen interactions, while in 1.7O_out_, the distribution appears more uniform. For 0.9Cl, an irregular distribution and asymmetric scattering of water molecules is observed due to subnanometer confinement and weaker water–Cl interactions. The radial density for the pores > 1 nm shows pronounced hydration shells, whereas 0.9Cl decays monotonically from the pore axis with no shell structure (Figure 2e). Overall, the pore water keeps the bulk 2.8 Å first-neighbor hydrogen bond but loses tetrahedral order with increasing confinement. This disorder is modulated by pore chemistry, where 1.6O and 1.7O_out_ retain more order than 0.9Cl due to the ether oxygens forming hydrogen bonds with the water molecules. This confinement-dependent structuring parallels water in silica nanopores, where bulk-liquid-like water is absent below approximately 2 nm and interfacial structuring dominates the pore;^75^ our sub-2 nm systems (0.9–1.7 nm) likewise show confinement-driven deviations from bulk order rather than a simple monotonic size trend, with pore chemistry (Cl vs ether O) modulating the degree of structuring within that regime.

### Proton Transport

Diffusion and permeability are directly related through the sorption coefficient;^76^ thus, we correlate theoretical diffusion with experimentally measured permeability. The transport experiments were performed using a Teflon test cell that consisted of two compartments, separated by the tested membrane, containing the feed and stripping solutions, respectively. The initial feed solution was 0.1 M HCl aqueous solution, and the stripping solution was 0.1 M aqueous solution of NaCl. Further details and proton permeability results are presented in the SI: Transport Experiments and Table S4. PECH75 exhibits a permeability of 0.55 ± 0.01 × 10^–6^ cm^2^/s, compared to 2.59 ± 0.05 × 10^–6^ cm^2^/s for Nafion 117. Theoretically, we characterize the dependence of water ordering on proton transport and explore confinement-pore functionality effects across structural models by simulating individual proton transport events.

The free-energy barriers for ten single proton-transfer events were computed with blue-moon-ensemble-constrained ab initio molecular dynamics at the PBE-D3 level^77^ and are reported in the SI (Figure 8). For statistical robustness, we report proton transport characterization along 5 independent 1 ns revPBE-D3, NVT MACE-MD trajectories, at 300 K and 0.5 fs time step, for twice the membrane column length (Figure 3).

**Figure 3:**
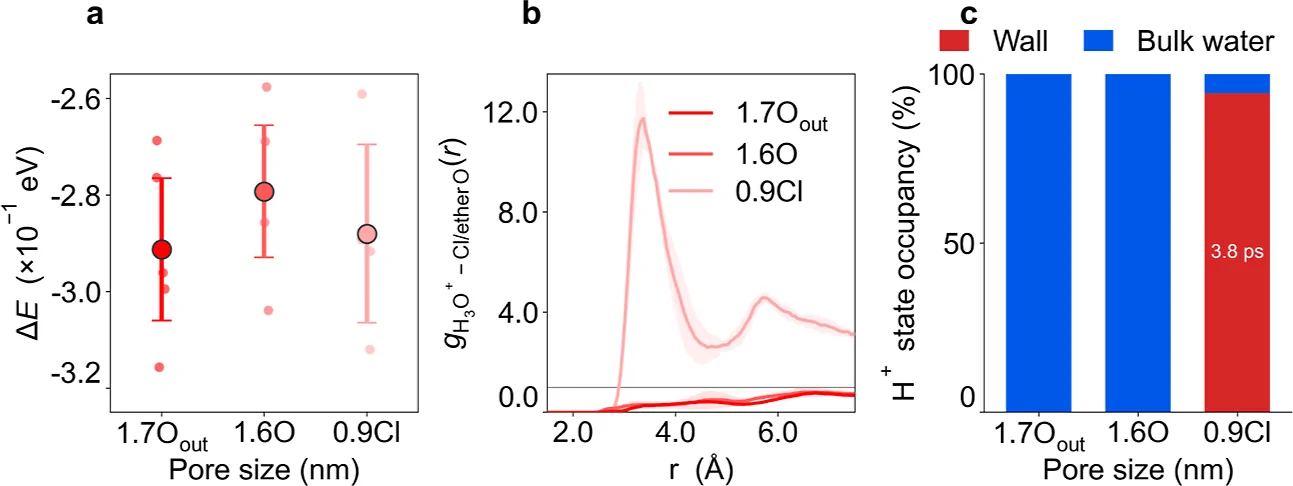
Proton-transfer mechanism in the three pores (5 independent MACE-MDs, each 1 ns atrevPBE-D3, 300 K, *NVT*, time step 0.5 fs at 2x membrane column length). (a) Proton-transfer energy-gap asymmetry Δ*E* = *E*(donor)–*E*(acceptor) per pore, from around 800 proton-transfer events sampled per system. (b) Radial distribution function between the excess proton (H_3_O^+^) and each pore’s wall group. (c) Fraction of the trajectory the excess proton (H^+^) spends trapped at the wall versus in central bulk water. The Δ*E* remains statistically identical across pores (−0.28 to −0.29 eV). The RDF peak is at 3.4 Å (*g* ≈ 12) for 0.9Cl, which is wall-bound 94% of the time (mean wall dwell: 3.8 ps).

The proton-transfer energy-gap asymmetry Δ*E* = *E*(donor)–*E*(acceptor), sampled over ≈ 800 proton-transfer events per system, remained uniform across all three pores (−0.28 to −0.29 eV), where about 88% of events remained donor-favored (Figure 3a).

The hydronium-to-wall RDF shows the proton binds the wall only in 0.9Cl, giving a sharp peak at 3.4 Å (*g* ≈ 12, Figure 3b). The ether-lined 1.6O and1.7O_out_ pores remain proton-inert (*g* < 1). In 0.9Cl, the proton is trapped at the Cl wall for 94% of the trajectory with a mean wall dwell of 3.8 ps, whereas in 1.6O and 1.7O_out_, it transports via the central bulk water essentially all the time without wall trapping (Figure 3c). In 1.6O and 1.7O_out_, the water hydrogen bonding to the ether oxygens (Figure 2b) keeps a connected central network that relays the proton, whereas the Cl in 0.9Cl accepts no such bond, disorders the water, and traps the proton. Proton transport is therefore relayed by the connected central water network, whereas under subnm confinement, the pore wall (Cl) shapes the interfacial water and captures the proton.

### Pore Design and Proton Transfer Mechanism

Subnanometer confinement and local chemistry both influence proton transport, evidenced by the contrasting behaviors of 1.6O and 0.9Cl. To further probe this interplay, we tuned the pore chemistry of 0.9Cl to investigate whether a favorable chemical environment can compete with the disordering effects of confinement and potentially improve the proton transfer dynamics. Keeping the pore size constant, we replaced the Cl atoms in 0.9Cl with OH groups (0.9OH), experimentally referred to as linear polyglycidol (LPG).^78^ It originates from glycidol, traditionally made by epoxidizing allyl alcohol with tungstic acid and peroxyacetic acid, thus relying on volatile oxidants and generating toxic byproducts.^79^ By contrast, a one-pot conversion of glycerol,^80^ a nontoxic, biodegradable coproduct of biodiesel, yields glycidol under mild conditions.^81^ This redesign avoids hazardous reagents and achieves bulk yields during the production of biodiesel from the transesterification of plant oils and animal fats. The structures of both 0.9Cl and 0.9OH are presented in Figures 4 and S9.

**Figure 4:**
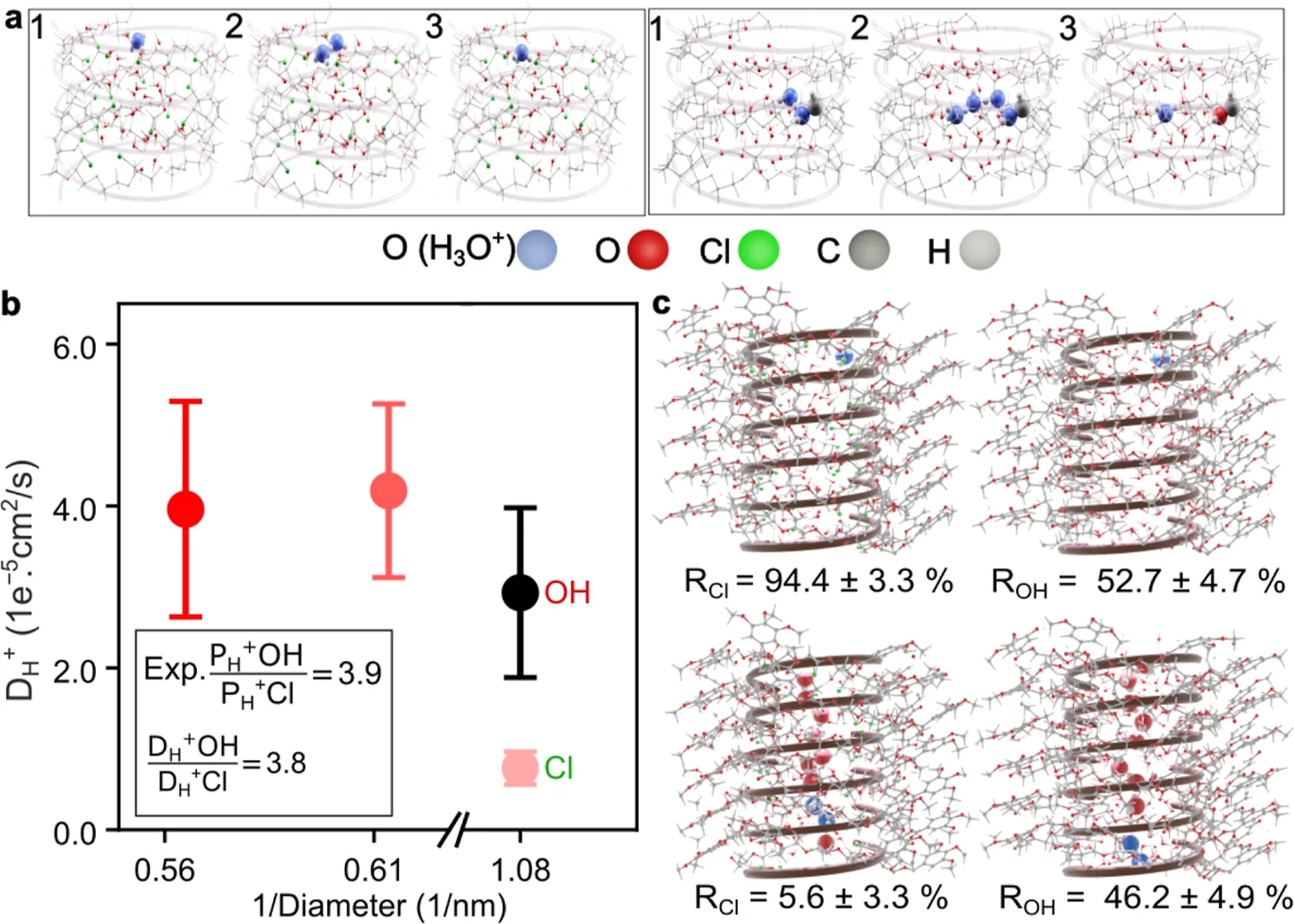
Diffusion characterization (5 independent MACE-MDs, each 1 ns at revPBE-D3, 300 K, *NVT*, timestep 0.5 fs at 2x membrane column length). (a) Proton-transfer mechanism from MD snapshots for 0.9Cl (left) and 0.9OH (right). In 0.9Cl, the proton is transferred to a nearby water via a Zundel ion, while in 0.9OH, it transfers via a Zundel ion followed by an Eigen ion in which the OH oxygen participates. (b) Proton diffusion coefficient *D*_H_+ against inverse pore diameter for all systems, from five independent 1 ns MACE-MD trajectories (dots average, bars standard deviation). Insets give the computed OH/Cl diffusion ratio $\left(D_{H^{+}}^{OH}/D_{H^{+}}^{Cl}=3.8\right)$ and the measured OH/Cl proton-permeability ratio $\left(P_{H^{+}}^{OH}/P_{H^{+}}^{Cl}=3.9\right)$ . (c) Excess-proton residence at the pore wall versus in the central water for 0.9Cl and 0.9OH: *R*_Cl_ = 94.4 ± 3.3% at the wall against 5.6 ± 3.3% central and *R*_OH_ = 52.7 ± 4.7% against 46.2 ± 4.9%. Blue spheres indicate H_3_O^+^ oxygens.

Considering the MD snapshots, OH groups directly participate in proton transfer by first forming a Zundel ion (H_5_O_2^+^_) with the nearby hydronium (H_3_O^+^) and then transferring the proton via an Eigen ion (H_9_O_4_^+^) (Figure 4a). On the contrary, in the 0.9Cl, the proton diffuses via a Zundel ion only involving water molecules. Hence, replacing Cl with OH modulates the proton-transfer landscape by increasing the number of transfer sites at the pore wall.

The proton diffusion coefficient (*D*_H_+), using the Einstein equation, is shown in Figure 4b. The diffusion coefficient increases with pore diameter, highest in the ether pores and lowest in the subnm pores. At the fixed 0.9 nm size, replacing Cl with OH raises *D*_H_+ 3.8-fold (Figure 4b), close to the larger ether pores and matching the 3.9-fold ratio of the measured OH/Cl proton permeabilities (Table S4). Although 0.9OH exhibits lower diffusion coefficients relative to Nafion at λ ≃ 20, it achieves comparable performance at significantly lower hydration levels (λ ≈ 2). Experimentally, approximately 10-fold lower water uptake for the equivalent LPG8 was observed compared to Nafion 117, therefore mitigating swelling effects (Table S4). The efficient proton transport in 0.9OH is facilitated by the wall keeping the near-pore water connected. The water hydrogen-bond coordination near the wall rises from 3.1 in 0.9Cl to 3.8 in 0.9OH (Figure S9a). The excess proton in turn contacts the hydroxyl oxygen (O*) at 2.5 Å, closer than the 3.4 Å chloride contact (Figure S9b).

A spanning hydrogen-bonded water wire is present in every pore during the MACE-MDs (Figure S10). The residence times show that in 0.9Cl the proton is trapped on the Cl for 94% of the trajectory (mean wall dwell: 3.8 ps) through electrostatic binding of the intact hydronium, which accounts for its low diffusion coefficient (Figure 4c). In 0.9OH, the hydroxyl holds the proton only transiently (52.7%, mean wall dwell: 0.33 ps) and diffuses faster. All three transport conclusions remain consistent at the PBE-D3 level (SI: Comparison with the PBE-D3 model).

Hence, the pore chemical environment modulates the proton-transfer landscape by influencing water order and availability of transfer sites at the pore wall, with narrower 0.9OH nm pores exhibiting enhanced proton conductivity due to more favorable diffusion pathways. Future modifications could involve altering OH group composition and blending with Cl groups to modulate the hydrogen-bond networks and proton transport. Exploring alternative functional moieties may further enhance the hydration control and proton diffusion.

## Conclusions

We modeled a low-cost liquid crystalline PECH-based proton exchange membrane using DFT and MACE molecular dynamics to elucidate structural configurations, water dynamics, and proton transport. Three optimized models exhibit distinct pore architectures: 0.9Cl with intrapore Cl atoms and 1.6O and 1.7O_out_ featuring inward- and outward-oriented ether oxygens, respectively. Individual proton transfer free-energy barriers were characterized using blue-moon-ensemble-constrained ab initio molecular dynamics, while extended MACE-MD simulations enabled quantification of cooperative transport properties and diffusion coefficients. Pore chemistry dominates transport behavior regardless of confinement, with the transfer energetics uniform across pores and water order tracking whether the hydrogen-bond network stays connected. Two distinct transport regimes emerge in subnm pores: enhanced diffusion in the central pore region via “water-wires” versus hindered transport near functional groups. Leveraging these insights, we engineered 0.9OH (LPG) with a subnm pore containing OH groups, combining favorable chemistry with confinement. Unlike conventional hazardous routes, the LPG can be sustainably synthesized from glycerol, a biodiesel byproduct, via a mild process aligning with environmental principles. This redesign achieves superior water ordering and 3.8 times faster diffusion than 0.9Cl (PECH). Experimental and theoretical analyses demonstrate diffusion comparable to Nafion 117 at significantly lower hydration levels, effectively mitigating swelling and eliminating “forever-chemicals”. Our DFT-ML design framework rationalizes macroscopic transport through molecular architecture, enabling atomistic design of the next-generation low-cost, sustainable F-free membranes.

## Methods

### Experimental Setups

#### Polyepichlorohydrin (PECH) Modification

Polyepichlorohydrin (PECH) is a commercial polymer with a weight-average molecular weight (*M*_w_) of 8.52 × 10^5^ and a number-average molecular weight (*M*_*n*_) of 3.2 × 10^5^ provided by Aldrich and used as received.

In a 125 mL Schlenk flask under argon,1 g (0.01 mol) of PECH was dissolved in anhydrous THF (100 mL) by stirring overnight at room temperature. Over the resulting viscous solution, the necessary amounts of potassium-3,4,5-tris­[4-(*n*-dodecan-1-yloxy)­benzyloxy]­benzoate and tetrabutylammonium bromide (TBAB) were added, in that order, with inert atmospheric techniques. The mixture was heated at 65 °C and magnetically stirred for 8 days. The reaction mixture was then diluted with hot tetrahydrofuran (THF) to reach a total volume of 200 mL, and the solution was precipitated into 1 L of warm water. The copolymer obtained was dissolved in hot THF and precipitated again in boiling 96% ethanol twice (400 mL per gram of polymer). After the second precipitation, the rubbery polymer mass was collected and dried at 55 °C under a vacuum for 48 h. Table S1 shows experimental details, reaction conditions, and the yield and degree of modification for various experiments.

Quantification was carried out from the ^1^H NMR spectra by comparing the areas of the aromatic peaks between 7.4 and 6.8 ppm, the benzylic protons at 4.8 ppm, and the methylenic protons at 4.4 ppm with the broad signal between 4.0 and 3.5 ppm (Figure S1). The results agreed with those of comparative elemental analysis.

#### Linear Polyglycidol (LPG) Preparation and Modification

Linear poly­[(1-ethoxyethyl)­glycidyl ether] was prepared from (1-ethoxyethyl)­glycidyl ether according to Schmitz et al.^82^ Linear polyglycidol was prepared by subsequent removal of the acetal protecting groups of [linear (1-ethoxyethyl)­glycidyl ether] under acidic hydrolysis conditions.

The linear polyglycidol was successfully modified by TAP using carbodiimide-mediated Steglich esterification^83^ between the free hydroxyl groups of the linear polyglycidol and the carboxylic group of TAP (Figure S11): in a three-necked round-bottom flask, a solution of TAP (2 g, 2 mmol) was prepared by dissolving it into dry DMF (5 mL). Stoichiometric amounts of DCC and DMAP at 0 °C were allowed to stir for 30 min in an Ar atmosphere. The necessary amount of linear polyglycidol was dissolved into dry DMF (5 mL) and added dropwise to the above reaction mixture. The reaction mixture was allowed to stir for 2 days at room temperature under an Ar atmosphere. Afterward, the reaction mixture was precipitated in 500 mL of methanol. The precipitated product was dissolved into THF and reprecipitated in 500 mL of methanol. This precipitation was repeated 5 times in order to remove DCC, DMAP, free acid, and other impurities. After precipitation, the modified polymer was collected and dried at 40 °C in vacuo for 48 h. Different degrees of modifications and corresponding yields are given in Table S5.

#### Membrane Preparation, Orientation, and Characterization

Membranes were prepared by the immersion precipitation process, in which a homogeneous polymer solution in THF (30% w/w) was cast on a FEP (fluorinated ethylene propylene) sheet support and immersed in a bath of Milli-Q water. The solvent diffused into the precipitation bath, while the nonsolvent (water) diffused into the cast film. After a time in which the solvent and the nonsolvent were exchanged, the polymer solution (wet film) became thermodynamically unstable, and demixing took place. Finally, a solid polymer membrane was formed with an asymmetric structure. Afterward, the membrane was dried overnight at room temperature and subsequently used for the thermal orientation process. The polymer membrane along with the FEP sheet was mounted on a hot stage (Linkam TP92), and it was heated above the copolymer clearing temperature; then, it was allowed to cool slowly (0.5 °C/min) down to room temperature. Afterward, the membrane was kept at room temperature for approximately 1 h, and then it was separated from the FEP sheet and obtained as an intact, uniform membrane with a thickness around 200 μm.

A JEOL F200 TEM ColdFEG operated at 200 kV was used for the transmission electron microscopy characterization. HR-TEM images were acquired with a Gatan OneView camera, a CMOS-based and optical fiber-coupled detector of 4096 by 4096 pixels. The Gatan Digital Micrograph program was used to process the TEM images. Cross sections (thickness 60–70 nm) of the PECH-75 oriented membranes were obtained by cryocutting at −55 °C with a Leica UC Enuity Ultracryomicrotome. The specimens were then differentially stained by exposing them on a support grid to RuO_4_ vapors for 2 h before analyzing.

### Computational Details

#### Density Functional Theory and Ab Initio Molecular Dynamics

We performed density functional theory (DFT) calculations using the Vienna Ab Initio Simulation Package (VASP) version 6.5.1.^84^ Core electrons were represented by projector-augmented wave (PAW) pseudopotentials,^85^ and the valence electrons were expanded in plane waves. We employed three exchange-correlation functionals: the generalized-gradient Perdew–Burke–Ernzerhof (PBE)^86^ and revised PBE (revPBE)^87^ functionals, each with the D3 dispersion correction of Grimme et al.^88^ (denoted PBE-D3 and revPBE-D3), and the meta-generalized-gradient r2SCAN functional.^89^ A plane-wave kinetic energy cutoff of 450 eV was used for PBE-D3 and revPBE-D3 and 600 eV for r2SCAN, the higher cutoff being required for the meta-GGA. Gamma-only *k*-point sampling and Gaussian smearing with a 0.05 eV smearing width were used. The electronic convergence criterion was at least 10^–6^ eV, and the force threshold for ionic convergence was set to 0.02 eV/Å. PBE-D3, revPBE-D3, and r2SCAN were used to generate the training and validation data for the MACE multihead model and to benchmark the sensitivity of the water structure to the choice of functional. The simulation box was extended to a supercell of three disks (cell size: 32 Å × 32 Å × 13 Å, atom count = 1197), and the entire structure was allowed to relax. All simulations used a time step of 0.5 fs and a Nose–Hoover thermostat^90–92^ at 300 K with symmetry operations disabled. The atomic mass of deuterium was used instead of hydrogen. The electronic convergence threshold was set to 10^–5^ eV. A fixed OH^–^ ion and a mobile excess proton were added to the simulation cell to ensure electroneutrality. The proton-transfer free-energy barriers were calculated at the PBE-D3 level using blue-moon-ensemble constrained molecular dynamics:^77,93^ the proton-transfer coordinate (the difference between the two O–H distances flanking the transferring proton) was advanced continuously by slow growth, and the mean constraint force was integrated along it to yield a free-energy profile rather than a static activation energy, with ten proton-transfer profiles averaged per system. Because these slow-growth barrier calculations are computationally expensive, the transport analysis instead quantifies the proton-transfer energy-gap asymmetry Δ*E* = *E*(donor)–*E*(acceptor) over around 800 transfer events per pore from the MACE-MD trajectories, a statistically robust measure that remains identical across pores (−0.28 to −0.29 eV). Active-learning loops built a multihead training set labeled at three DFT levels (PBE-D3, revPBE-D3, and r2SCAN), comprising 11,332 configurations in total (4,597/3,418/3,317 per head), for training of the MACE model.

### Model Construction

The monomer consists of a hydrophilic poly­(epichlorohydrin) chain, PECH, attached to the hydrophobic side group potassium 3,4,5-tris­[4-(*n*-dodecan-1-yloxy)­benzyloxy]­benzoate (Figure 1b).^68^ Since the hydrophobic side groups do not participate in proton transport, only PECH, where R groups were capped with H atoms, was considered as the model monomer. Based on the chloromethylrotation in the linear chain and the C–O–C bond angle, a total of three monomer conformations were considered (Figure 1a: top). Before building the membrane models, all three monomers were optimized in the gas phase with a 10 Å vacuum in all directions.

The columnar structure of the membrane forms by repeating vertically interconnected disks. Each disk is simulated by linking eight monomers to form a full 360° circle. The helical nature is introduced by translating each subsequent monomer in the *z*-direction. The translating distance is the disk height (4.5 Å) divided by the total number of monomers per disk (8). The simulation cell contained one disk, and the structure was periodically extended in the *x*, *y*, and *z* directions. This led to three membrane models, 0.9Cl, 1.6O, and 1.7O_out_, with the pore sizes of 0.93 nm, 1.63 nm, and 1.77 nm, respectively. All three models were optimized, i.e., both cell size and atomic positions, before adding the water molecules. The experimental XRD reveals the number of monomers per disk and the vertical distance between each disk.^68^

### Pore Solvation

Each model was filled, iteratively, with an increasing number of water molecules until the difference in adsorption energy per water molecule (Δ*E*_ads_ per water) exceeded the adsorption energy of the water dimer (Δ*E*_ads_ water dimer). When Δ*E*_ads_ per water surpasses the Δ*E*_ads_ water dimer (−0.2 eV), the membrane–water interaction becomes weaker than the water–water interaction. The number of water molecules required for 0.9Cl, 1.6O, and 1.7O_out_ is 13, 31, and 32, respectively. The theoretical water uptake (WU) is calculated by dividing the total mass of water by the mass of one polymer disk.

### Molecular Dynamics Simulations Using the MACE Potential

MACE models were trained using the MACE architecture (0.3.15).^71^ The MACE version used for training and inference corresponds to the GitHub repository https://github.com/ACEsuit/mace/releases/tag/v0.3.15 with CUDA 12.4. Full MACE training details are presented in the SI: MACE. The training set (with 12% randomly selected configurations held for validation) was generated using the Query by Committee (QbC) active learning approach.^94^ The 13 ps AIMD trajectory was randomly sampled (300 configurations) to initialize the active learning. The iterative process is terminated when fewer than 7% of the configurations display a force disagreement above 0.1 eV Å^–1^ and less than 0.7% exceed a maximal deviation of 0.15 eV Å^–1^. For the selected configurations, reference energies and forces are computed and labeled at three DFT levels (PBE-D3, revPBE-D3, and r2SCAN) to train a multihead MACE model, with 4,597/3,418/3,317 configurations per head (11,332 in total) and 835/421/403 configurations held out for validation, respectively. The model accuracy for energy, forces, and O–ORDF are presented in Figure S4. The production MACE-MDs were run on double the column length for each membrane, i.e., 6 rings (cell size = 32 × 32 × 26 Å^3^, atom count = 2739). The ASE Python package (MACE calculator) was used to perform MD simulations using the MACE model. The training/inference parameters and protocol details are presented in the SI: MACE.

## Supplementary Material



## Data Availability

DFT computational data in the ioChem-BD repository can be accessed using 10.19061/iochem-bd-1-381. The final multihead MACE machine-learning potential used for the molecular dynamics simulations is openly available at https://github.com/muhammad-saad-naeem/fluorine-free-membrane-mace-model.

## Notes

Experimental synthesis of the polyepichlorohydrin, modified PECH, and linear polyglycidol membranes; membrane preparation, orientation, and XRD characterization; proton-transport measurements and cost estimation; computational model construction, pore solvation, and water-uptake protocols; density functional theory, ab initio molecular dynamics, and MACE machine-learning potential methodology and validation; orientational and translational tetrahedral order, local structure index, and diffusion-coefficient analysis; comparison of the revPBE-D3 and PBE-D3 models covering water structure and proton transport; and supporting figures and tables (PDF)
