# Supplementary material for: Tuning Pore Confinement and Chemistry of Fluorine-Free Membranes
Source: J Am Chem Soc. 2026 Oct 9;148(41):45049–58. doi: 10.1021/jacs.6c15621 (PMC13693448; doi:10.1021/jacs.6c15621)
Supplement: Supplementary file 1 [file ja6c15621_si_001.pdf]

Supporting Information: Tuning pore confinement and chemistry of Fluorine-free membranes

Muhammad Saad Naeem,[*,†,‡] Zan Lian,[†] Jordi Guardià,[¶] Ranga Rohit Seemakurthi,[†] Pol Sanz-Berman,[†,‡] Marta Giamberini,[§] Ricard Garcia-Valls,[§] and Núria López[*,†]

†*Institute of Chemical Research of Catalonia (ICIQ-CERCA), The Barcelona Institute of Science and Technology, (BIST), Av. Països Catalans 16, Tarragona, 43007, Spain*

‡*Universitat Rovira i Virgili, Campus Sescelades, N4 Block, C. Marcel·lí Domingo 1, Tarragona, 43007, Spain*

¶*Department of Analytical Chemistry and Organic Chemistry, Universitat Rovira i Virgili, Carrer Marcel.lí Domingo 1, 43007 Tarragona Spain*

§*Department of Chemical Engineering, Universitat Rovira i Virgili, Av. Països Catalans, 26 43007 Tarragona Spain*

E-mail: saadmlk33@gmail.com,mnaeem@iciq.es; nlopez@iciq.es

Experimental

Synthesis

Polyepichlorohydrin (PECH)

Polyepichlorohydrin (PECH) is a commercial polymer (weight-average molecular weight (M_w) 8.52×10^5 and number-average molecular weight (M_n) 3.2×10^5) provided by Aldrich and used as received.

Modified PECH

In a 125-mL Schlenk flask under argon, 1 g (0.01 mol) of PECH was dissolved in anhydrous THF (100 mL) by stirring overnight at room temperature. Over the resulting viscous solution, the necessary amounts of potassium 3,4,5-tris[4-(n-dodecan-1-yloxy)benzyloxy]benzoate) and Tetrabutylammonium bromide (TBAB) were added, in this order, with inert atmospheric techniques. The mixture was heated at 65 °C, and magnetically stirred for 8 days. The reaction mixture was then diluted with hot tetrahydrofuran (THF) to reach a total volume of 200 mL, and the solution was precipitated into 1 L of warm water. The copolymer obtained was dissolved in hot THF and precipitated again in boiling 96% ethanol twice (400 mL per gram of polymer). After the second precipitation, the rubbery polymer mass was collected and dried at 55 °C under vacuum for 48 h. Table. S1 shows experimental details, reaction conditions, and the yield and degree of modification for various experiments.

Quantification was carried out from the ^{1}H NMR spectra by comparing the areas of the aromatic peaks between 7.4 and 6.8 ppm, the benzylic protons at 4.8 ppm, and the methylenic protons at 4.4 ppm with the broad signal between 4.0 and 3.5 ppm (Fig. S1). The results agreed with those of the comparative elemental analysis.

Linear polyglycidol (LPG) preparation and modification

Linear poly[(1-ethoxyethyl)glycidyl ether] was prepared from (1-ethoxyethyl)glycidyl ether according to Schmitz et al.[1] Linear polyglycidol was prepared by subsequent removal of the acetal protecting groups of [linear (1-ethoxyethyl)glycidyl ether] under acidic hydrolysis conditions.

The linear polyglycidol was successfully modified by TAP using carbodiimide mediated Steglich esterification[2] between the free hydroxyl groups of the linear polyglycidol and carboxylic group of TAP (Fig. S11): In three necked round bottom flask, solution of TAP (2 g, 2 mmol) was prepared by dissolving into dry DMF (5 mL). Stoichiometric amounts of DCC and DMAP at 0ºC were allowed to stir for 30 min in an Ar atmosphere. The necessary amount of linear polyglycidol was dissolved into dry DMF (5 ml) and added dropwise to the above reaction mixture. The reaction mixture was allowed to stir for 2 days at room temperature under Ar atmosphere. Afterwards, the reaction mixture was precipitated in 500 ml of methanol. Precipitated product was dissolved into THF and re-precipitated in 500 ml methanol. This precipitation was repeated for 5 times, in order to remove DCC, DMAP, free acid and other impurities. After precipitation, the modified polymer was collected and dried at 40°C in vacuo for 48 hours. Different degrees of modifications and corresponding yields are given in Table. S5.

Membrane preparation and orientation

Membranes were prepared by immersion precipitation process, in which a homogeneous polymer solution in THF (30% w/w) was cast on a FEP (fluorinated ethylene propylene) sheet support and immersed in a bath of Milli-Q water. The solvent diffused into the precipitation bath, while the non-solvent (water) diffused into the cast film. After a time in which the solvent and the non-solvent were exchanged, the polymer solution (wet film) became thermodynamically unstable and demixing took place. Finally, a solid polymer

membrane was formed with an asymmetric structure. Afterwards, the membrane was dried overnight at room temperature and subsequently used for thermal orientation process. The polymer membrane along with FEP sheet was mounted on a hot stage (Linkam TP92) and it was heated above the copolymer clearing temperature; then it was allowed to cool slowly (0.5°C/min) down to room temperature. Afterwards, the membrane was kept at room temperature for approximately 1 hour and then it was separated from the FEP sheet and obtained as an intact, uniform membrane with thickness around 200 μm.

XRD characterization

XRD measurements were made using a Bruker-AXS D8-Discover diffractometer equipped with a parallel incident beam (Göbel mirror), vertical θ-θ goniometer, XYZ motorized stage, and a GADDS (General Area Diffraction System). Samples were placed directly on the Si(510) sample holder for reflection analysis. An X-ray collimator system close to the sample allows one to analyze areas of 500 μm. The X-ray diffractometer was operated at 40 kV and 40 mA to generate CuKα radiation. The GADDS detector was a VÅNTEC-500 (silicon strip technology) placed at 30 cm from the sample. We collected one frame (2D XRD pattern) that covered, at such a distance, a range from 0.9° up to 11° 2θ and from 9.0° to 30.5° 2θ. The exposition time was 600 s per frame. The X-Ray diffractogram of oriented PECH75 is presented in Fig. S2.

Transport Experiments

The transport experiments were performed using a Teflon test cell that consisted of two compartments, separated by the tested membrane, containing the feed and the stripping solutions, respectively. The feed and the stripping volumes were 200 mL and the effective membrane area was 0.86 cm^2. For the proton transport experiments, the initial feed solution was 0.1 M HCl aqueous solution and the stripping solution 0.1 M aqueous solution of NaCl.

The pH of the stripping solution was measured every 10 s by an Orion Dual Star pH/ISE Multimeter. All experiments were repeated three times. The calculations of the proton permeability were carried out on the basis of the following equations:

Under steady-state conditions, proton flux was calculated by Fick's First Law:

$$J = -D\frac{dC}{dx} \approx \frac{D\Delta C}{l} \tag{S1}$$

where l (cm) is the membrane thickness and ΔC is the difference in proton concentration (mol L^{-1}) between the initial feed solution (C_0) and the final stripping solution. In our experimental conditions, C_0 was much greater than the final stripping concentration, so we considered $\Delta C \approx C_0$.

P is the proton permeability (cm^2 s^{-1}), defined as:

$$P = DS \tag{S2}$$

where D is the proton diffusion coefficient and S is the sorption equilibrium parameter.

The flux is related to the permeability coefficient p (cm s^{-1}), as:

$$J = p\Delta C \tag{S3}$$

$$p = \frac{DS}{l} \tag{S4}$$

The permeability coefficient can be described by the following equation:

$$\ln\left(1 - \frac{C_f}{C_0}\right) = -\frac{A}{V_f}pt \tag{S5}$$

where C_0 (mol L^{-1}) is the initial concentration of the feed solution and C_f (mol L^{-1}) is the

feed concentration calculated from the stripping solution at time t (s):

$$C_f = \frac{V_s}{V_f} C_s \tag{S6}$$

V_f is the feed volume (mL) and A is the actual membrane area (cm^2).

It should be noted that proton permeability (as determined by this test) is actually limited by Na^+ permeability, since it is a cation antiport transport mechanism. This was verified by comparing the Na^+ concentration in the feed compartment at the start-up time and at the end of the experiment with the increase of proton concentration in the stripping compartment consequent to its transport. Within experimental error, the variation was equal, with values of 11.43 (± 0.01) ppm for $\Delta[H^+]$ and 12 (± 1) ppm for $\Delta[Na^+]$, thus confirming a cation antiport mechanism in the oriented membranes. Different permeability values are obtained in case LiCl or KCl are used in the stripping compartment, in line with the different characteristics of the cations.

Cost Estimation

Based on the lab-scale pricing and yields, the PECH based membrane is estimated at 2-3 times the current cost of Nafion 117 (€3200–3350 per m^2 for PECH vs €1350–2100 per m^2 for Nafion 117). However, this analysis does not account for the cost reduction at scale and elimination of continuous characterization (e.g., NMR, XRD).

At a loading of 50 mg/cm^2, a 1m^2 of membrane requires 500g of polymer. For the highest modification degree (PECH75), the PECH/TAP ratio of 11.8 wt% (or 7.3 wt% under worst-case yields) corresponds to 59 g PECH and 441g TAP. With PECH (MW = 700,000) priced at €0,32 per g and TAP at€7.2 per g, TAP contributes to the 99% of the total material cost. Reduction in TAP loading by either lower degrees of modification to PECH or using LPG backbone with pendant –OH groups instead of chlorine, (requiring TAP loading as low

as 11%) could further lower the cost without compromising proton permeability.

All material prices are based on Sigma Aldrich's 2024 data except for TAP. The current cost of TAP was estimated at €6 per g in 2016 (€3 for materials plus €3 for analytical costs) with annual inflation adjustment of 1.8%.

Computational Details

Model Definition and Construction

Model Construction

The monomer consists of a hydrophilic poly(epichlorohydrin) chain, PECH, attached to the hydrophobic side group potassium 3,4,5-tris[4-(n-dodecan-1-yloxy)benzyloxy]benzoate (Fig. **??** a: top).[3] Since the hydrophobic side groups do not participate in proton transport, only PECH, where R groups were capped with H atoms, was considered as the model monomer. Based on the chloromethyl rotation in the linear chain and the C-O-C bond angle, a total of three monomer conformations were considered (Fig. **??** a: bottom). Before building the membrane models, all three monomers were optimized in gas phase with 10 Å vacuum in all directions.

The columnar structure of the membrane forms by repeating vertically interconnected disks. Each disk is simulated by linking eight monomers to form a full 360° circle. The helical nature is introduced by declining each subsequent monomer in the z-direction. The declining distance is the disk height (4.5 Å) divided by the total number of monomers per disk (8). The simulation cell contained one disk, and the structure was periodically extended in x, y, and z directions. This led to three membrane models 0.9Cl, 1.6O, and $1.7O_{out}$ with pore sizes of 0.93 nm, 1.63 nm, and 1.77 nm, respectively. All three models were optimized before the addition of the water molecules.

The optimized simulation cell laterally measured to be approximately 3.2 nm. This size was validated against High-Resolution Transmission Electron Microscopy (HR-TEM) imaging of PECH75 membranes, where samples were prepared by perpendicular cryocutting at -80°C and RuO_4 vapor treatment for 8 minutes to enhance contrast. The HR-TEM images revealed parallel aligned columnar structures (Fig. S3) with a width of 4.5 nm, measured by Fourier transform analysis and consistent with X-ray diffraction measurements.[4] The 1.3 nm difference between the simulation and the experimental dimensions is due to the exclusion of mesogenic groups from the computational model to enhance the computational efficiency.

Detailed experimental insights including XRD results that reveal the vertical distance between each disk are presented in.[3–5]

Pore solvation

To build the computational model, each system was filled, iteratively, with an increasing number of water molecules until the difference in adsorption energy per water molecule (ΔE_{ads} per water) exceeded the adsorption energy of the water dimer (ΔE_{ads} water dimer). When ΔE_{ads} per water surpasses the ΔE_{ads} water dimer (-0.20 eV), the membrane-water interaction becomes weaker than the water-water interaction. The number of water molecules required for 0.9Cl, 1.6O, and 1.7O_{out} is 13, 31, and 32, respectively. The theoretical water uptake (WU) is calculated by dividing the total mass of water by the mass of one polymer disk.

Water Uptake

The membrane model is built by vertically repeating a ring-shaped helical chain composed of 8 PECH monomer units. The monomer considered for the model construction is lighter due to the missing bulky mesogenic groups. Thus, for calculating the theoretical and experimental water uptake, the molecular weight of the real polymer is considered. The 8 units per

ring are divided according to the experimental PECH75 system i.e. 75% modified with the mesogenic groups. Hence, 6 units with the mesogenic groups + 2 units bearing the chlorine = 6131 g. The number of water molecules per ring for each model is used for the numerator. Since the model is a single vertical column, the theoretical water uptake is comparable to the un-oriented experimental membranes.

$$\text{Water uptake } \% = \frac{\text{MW for water molecules per ring}}{\text{MW for the ring}} \tag{S7}$$

where:

$$\text{MW} = \text{Molecular weight}$$

$$1 \text{ Ring} = 8 \text{ PECH monomer units}$$

Computational Methodologies

Density Functional Theory

We performed density functional theory (DFT) calculations using the Vienna Ab Initio Simulation Package (VASP) version 6.5.1.[6] Core electrons were represented by projector-augmented wave (PAW) pseudopotentials,[7] and the valence electrons were expanded in plane waves. We employed three exchange-correlation functionals: the generalized-gradient Perdew-Burke-Ernzerhof (PBE)[8] and revised PBE (revPBE)[9] functionals, each with the D3 dispersion correction of Grimme et al.[10] (denoted PBE-D3 and revPBE-D3), and the meta-generalized-gradient r2SCAN functional.[11] A plane-wave kinetic energy cutoff of 450 eV was used for PBE-D3 and revPBE-D3, and 600 eV for r2SCAN, the higher cutoff being required for the meta-GGA. Gamma-only k-point sampling (cell size 32Åx32Åx13Å, atom count = 1197) and Gaussian smearing with a 0.05 eV smearing width were used. The electronic convergence criterion was at least 10^{-6} eV, and the force threshold for ionic convergence

was set to 0.02 eV/Å. PBE-D3, revPBE-D3 and r2SCAN were used to generate the training and validation data for the multihead MACE model and to benchmark the sensitivity of the water structure to the choice of functional.

Ab initio molecular dynamics simulations

The simulation box was extended to a supercell of three disks, and the entire structure was allowed to relax. All simulations used a time step of 0.5 fs and a Nose-Hoover thermostat[12–14] at 300 K with symmetry operations disabled. The atomic mass of deuterium was used instead of hydrogen. The electronic convergence threshold was set to 10^{-5} eV. A fixed OH^- ion and a mobile excess proton were added to the simulation cell to ensure electroneutrality. The proton-transfer free-energy barriers were calculated at the PBE-D3 level using blue-moon-ensemble constrained molecular dynamics:[15,16] the proton-transfer coordinate (the difference between the two O-H distances flanking the transferring proton) was advanced continuously by slow growth and the mean constraint force integrated along it to yield a free-energy profile rather than a static activation energy. Each run entails a single proton-transfer event in which the excess proton, driven along the coordinate, transfers from the hydronium to a neighboring water molecule; ten proton-transfer profiles were averaged to obtain the mean free-energy barriers reported. Because these slow-growth barrier calculations are computationally expensive, the transport analysis in the main text instead quantifies the proton-transfer energy-gap asymmetry $\Delta E = E(\text{donor}) - E(\text{acceptor})$ over $\approx$800 transfer events per pore from the MACE-MD trajectories, a statistically robust measure that remains identical across pores (-0.28 to -0.29 eV). The functional comparison of bulk-water structure (Fig. S6) used a separate cubic box of 64 water molecules simulated under the same NVT protocol for 80 ps each for PBE-D3, revPBE-D3, and r2SCAN.

Molecular dynamics simulations using the MACE potential

MACE models were trained using the MACE architecture (0.3.15).[17] The MACE version used for training and inference corresponds to the GitHub repository `https://github.com/ACEsuit/mace/releases/tag/v0.3.15` with CUDA 12.4. The model consists of two invariant hidden layers with 64 channels (64x0e as implemented in MACE), and correlation order 3 (body order 4). Each layer utilized 8 radial and 5 basis functions with a radial cutoff of 6 Å, resulting in a total receptive field of 12.0 Å per atom. The model was trained using the AMSGrad optimizer[18] with an exponential moving average decay of 0.99. The learning rate was initialized at 0.008, and reduced by a factor of 0.7 after 3 epochs of validation loss plateau. The training continued for a maximum of 400 epochs with early stopping triggered after 11 epochs of no validation loss improvement. The training data was fed with a batch size of 6 with float32 precision in CUDA implementation. Training proceeded using Stochastic Weight Averaging (SWA)[19] with a weighted sum of mean squared errors energy-forces loss function: initially with energy and forces weights at 100 and 1200 respectively, followed by a second stage starting from last 20% epochs (learning rate = 0.001) with adjusted weights of 2000 and 100, respectively. Weights and Biases (wandb) optimized the hyperparameter choices for minimal validation loss using the Hyperband based Bayesian approach.[20] The isolated atomic energies are computed using least square regression as implemented in MACE.

The training set (with 12% randomly selected configurations held for validation) was generated using Query by Committee (QbC) active learning approach.[21] The initial dataset contained 300 configurations (100 for each membrane) randomly sampled from a 13ps AIMD trajectory with a timestep of 0.5 fs at 300K. The dataset is iteratively augmented based on energy and forces disagreements (each given equal weight) among a committee of five models. The best-performing model from the committee is used to propagate a 30-ps molecular dynamics trajectory with a timestep of 0.5fs, during which the temperature is ramped continuously from 200K to 420K. The configurations and their corresponding energy and forces

were sampled every 100 MD steps. In every iteration, 200 structures with the highest disagreement are added to the training set. The iterative process is terminated when fewer than 7% of the configurations display a force disagreement above 0.1 $eV\text{Å}^{-1}$ and less than 0.7% exceed a maximal deviation of 0.15 $eV\text{Å}^{-1}$. For the selected configurations in each active learning iteration, reference energies and forces are computed. The production model is a single multihead MACE model with three functional-specific readout heads (PBE-D3, revPBE-D3 and r2SCAN) sharing a common representation, trained jointly from the start on 4,597 (PBE-D3), 3,418 (revPBE-D3) and 3,317 (r2SCAN) configurations (11,332 in total), with 835, 421 and 403 configurations held out for validation, respectively. The transport properties reported in the main text use the revPBE-D3 head; the PBE-D3 and r2SCAN heads bracket the sensitivity of the water structure to the choice of functional (Fig. S4). The composition of the final dataset in terms of different samples and temperature is reported in Fig. S12.

To ensure an unbiased and diverse sampling of water structures, the initial configurations for the 30-ps MACE-MD simulations were prepared by solvating each membrane at varying hydration levels (0.9-2.0 g/cm^3 across all pore sizes) and water molecules randomly initialized using the packing model[22] implemented in PACKMOL.[23]

The bulk-water O-O radial distribution function benchmark against the reference DFT (Fig. S4) was computed from independent 80 ps MACE-MD simulations run with each functional head. The ASE Python package (MACE calculator) was used to perform MD simulations using the MACE model. Production MACE-MD runs used doubled column lengths (6 rings) with cell dimensions of 32Å×32Å×26Å containing 2739 atoms. The first 10 ps of each trajectory were excluded as equilibration.

Beyond bulk water, we verified that the potential also reproduces the structure of the confined membrane water. The water-oxygen O–O RDF inside the 0.9Cl pore was compared against a DFT *ab initio* MD reference for both revPBE-D3 head and PBE-D3 (Fig. S5).

MACE reproduces the DFT first-peak height to within the statistical noise (revPBE-D3 +0.8%; PBE-D3 +0.4%) and the peak position to within 0.03 Å. This level of agreement sits within the few-percent band established for machine-learning potentials of liquid water, for which the first-peak position is reproduced against the reference DFT to better than 0.05 Å[24,25] and the first-peak height fluctuates around the reference by roughly 2%.[26–28]

Tuning the pore

The Cl functional groups in 0.9Cl were replaced with OH groups. Following geometric optimization (relaxing atomic positions), the system was used for production MACE-MD simulations.

Analysis

Orientational Tetrahedral Order

It considers the local order based on the angular arrangement between the closest water oxygen neighbors and is denoted by q.[29,30] It varies from 0, characteristic of ideal gas to 1 for a regular tetrahedron. For the calculation, it considers the 4 nearest water oxygens around a reference oxygen.

$$q = 1 - \frac{3}{8}\sum_{j=1}^{3}\sum_{k=j+1}^{4}\left(\cos\psi_{jk} + \frac{1}{3}\right)^2 \tag{S8}$$

The ψ_{jk} is the angle between the two nearest water oxygen neighbors (j and k) and the oxygen atom of interest. The q closer to one indicates a higher local order in the water molecules.

The bulk-water orientational tetrahedral order q computed at the PBE-D3 level is unimodal and peaks at $q \approx 0.90$ (mean $q = 0.84$), reflecting the well-documented over-

structuring of PBE-family water; Chen et al. report $q = 0.83$ for PBE and describe it as an overly tetrahedral liquid.[31] Recomputing q with the revPBE-D3 functional gives $q = 0.70$, in close agreement with the value $q \approx 0.67$ reported for liquid water at ambient conditions (TIP4P/2005).[32] r2SCAN is intermediate ($q = 0.76$). The elevated tetrahedral order in the bulk-water reference therefore originates from the exchange-correlation functional rather than from the analysis, and the dispersion-corrected revPBE-D3 recovers the accepted liquid-water order (Fig. S6).

Diffusion Coefficient

The self-diffusion coefficient is computed by fitting the mean square displacement (MSD) as a function of the lag time using the Einstein relation. The lag time of 0.1 to 0.5 times the simulation time is used to avoid the ballistic trajectory in the short-time region.[33]

The self-diffusion coefficient D is given by:

$$D = \frac{1}{2d} \lim_{t \to \infty} \frac{\left\langle [r(t_0 + t) - r(t_0)]^2 \right\rangle}{t} \tag{S9}$$

where D is the self-diffusion coefficient, d is the dimensionality of the system, t is time, t_0 is the initial time, and $\left\langle [r(t_0 + t) - r(t_0)]^2 \right\rangle$ is the mean squared displacement (MSD).

No finite-size correction was applied to the diffusion coefficients reported here. The Yeh-Hummer correction[34] adds a term $\xi k_B T/(6\pi\eta L)$ that is derived for a species diffusing isotropically through bulk solvent in a periodic box. In the systems studied here a proton is confined to a quasi-one-dimensional pore whose walls are an explicit part of the simulation cell. The four systems share the same cell dimensions and the same pore geometry, so a common residual finite-size contribution exists and cancels in the functional-group ratios we quote.

Comparison of revPBE-D3 and PBE-D3

Water structure

No second solvation shell near 4.5 Å in any pore was found. 0.9Cl is the most disordered pore at both levels. The water coordination deficit at the chloride wall, and the contrast between the directed ether-oxygen contact and the diffuse chloride contact, hold at both levels (Figs. S13 and S14 at PBE-D3, against Fig. S9 at revPBE-D3).

We found PBE-D3 over-structures bulk water, $q = 0.843 \pm 0.002$ against 0.695 ± 0.004 at revPBE-D3 and ≈ 0.67 for liquid water.[32] The ranking of the pores is unchanged, but confinement now lowers q relative to bulk.

Proton transport

The excess proton stays trapped at the chloride wall and is held only transiently at the hydroxyl wall. The two regimes persist, wall-trapped against relayed by the central water wire. Replacing Cl by OH at fixed 0.9 nm pore size raises D_{H^+}. The computed OH/Cl ratio moves from 2.4 to 3.8, against a measured permeability ratio of 3.9 (Table. S6).

The highest D_{H^+} is now found in the ether pores, where the original PBE-D3 result placed it in 0.9OH (Fig. S15b). The ether pores are the more ordered ones and show no wall trapping, whereas 0.9Cl holds the proton for 94% of the trajectory. Pore size and wall chemistry are therefore separated, and the chemistry comparison is made at fixed 0.9 nm. The residence contrast between 0.9Cl and 0.9OH is present at both levels (Fig. S15c at PBE-D3, Fig. **??**c at revPBE-D3), and is larger at revPBE-D3 (Table. S6).

Supporting Figures

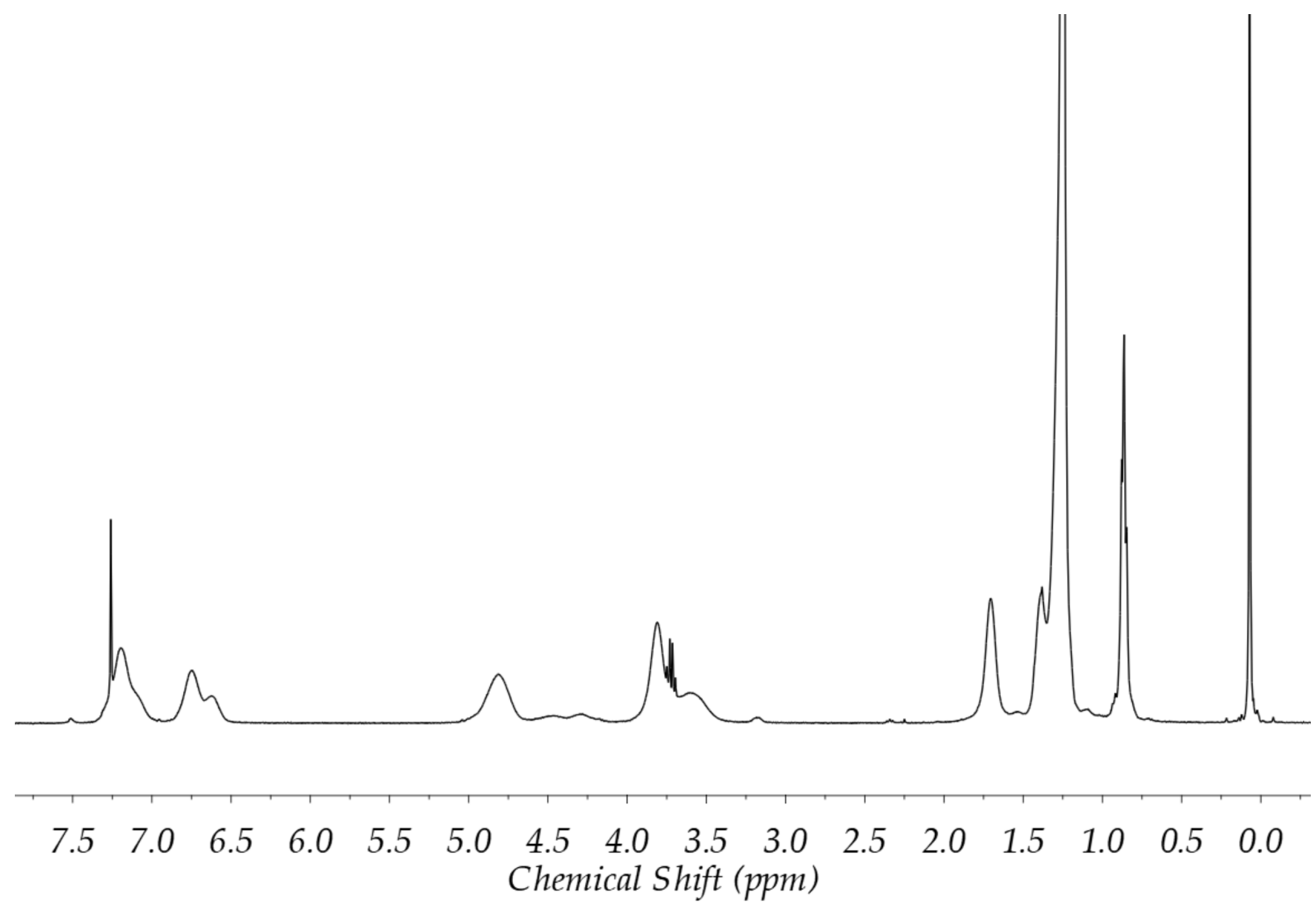

Figure S1: ^{1}H NMR spectrum of p-(n-dodecan-1-yloxy)benzyl alcohol.

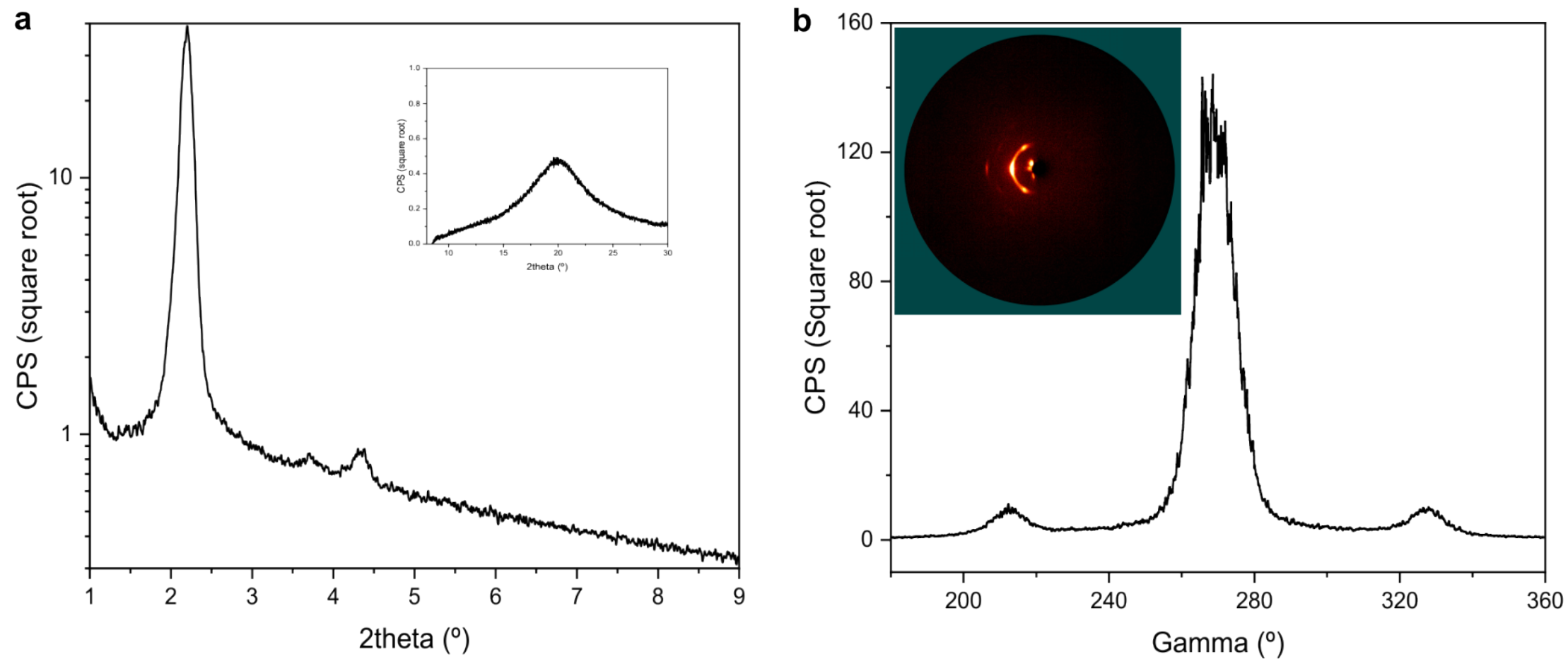

Figure S2: (a) X-Ray diffractogram of oriented PECH75. (b) The azimuthal scan of the reflection at $2\theta = 2.2^{\circ}$. Inset: Debye pattern of PECH75 oriented membrane

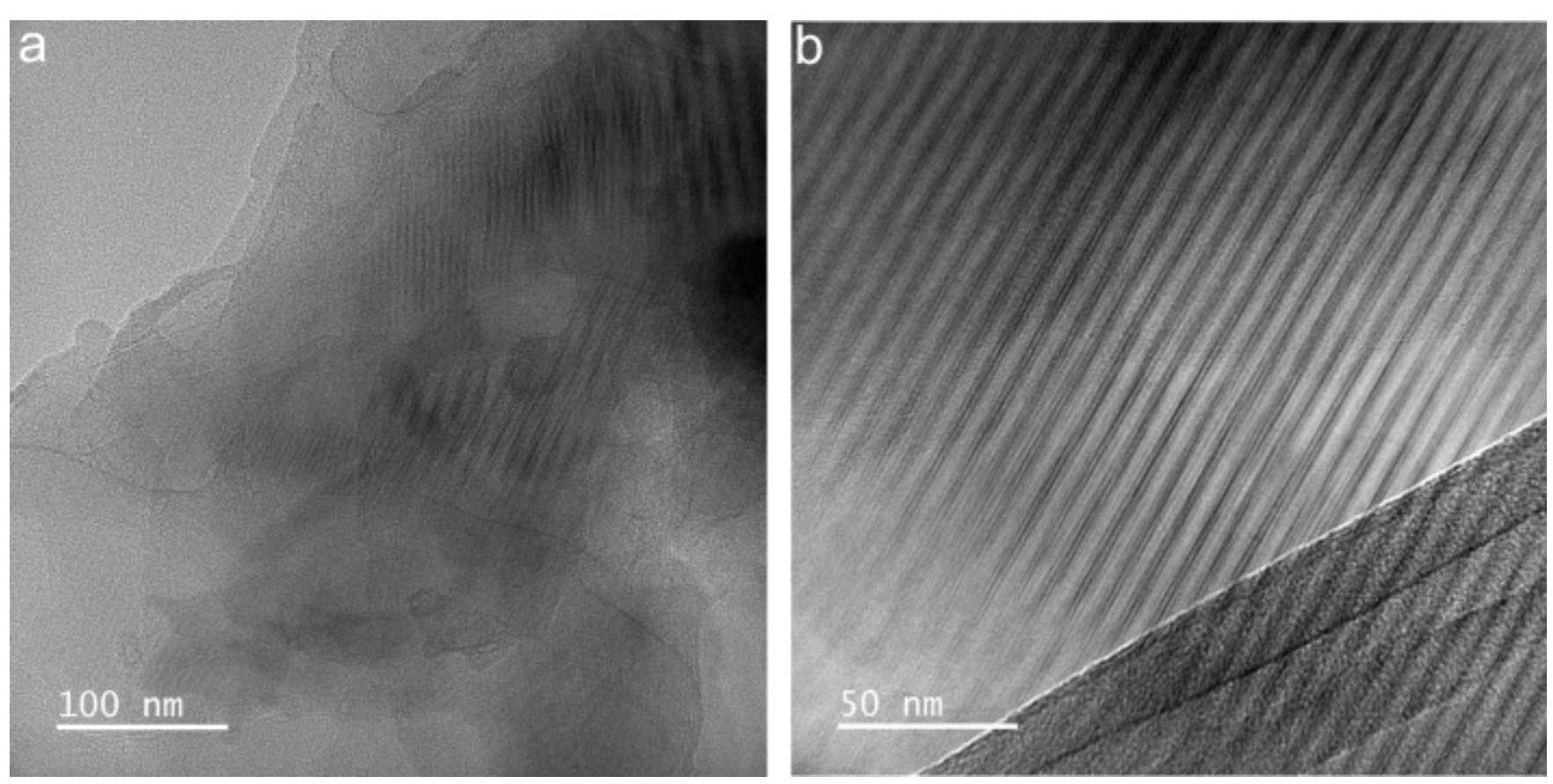

Figure S3: HR-TEM images of the oriented PECH75 membrane. (a) and (b) display the parallel alignment of ion columns. The column width, measured as 4.5 nm using Fourier transform analysis, accounting for the exclusion of mesogenic groups from the model, is consistent with the model simulation cell i.e 3.2 nm.

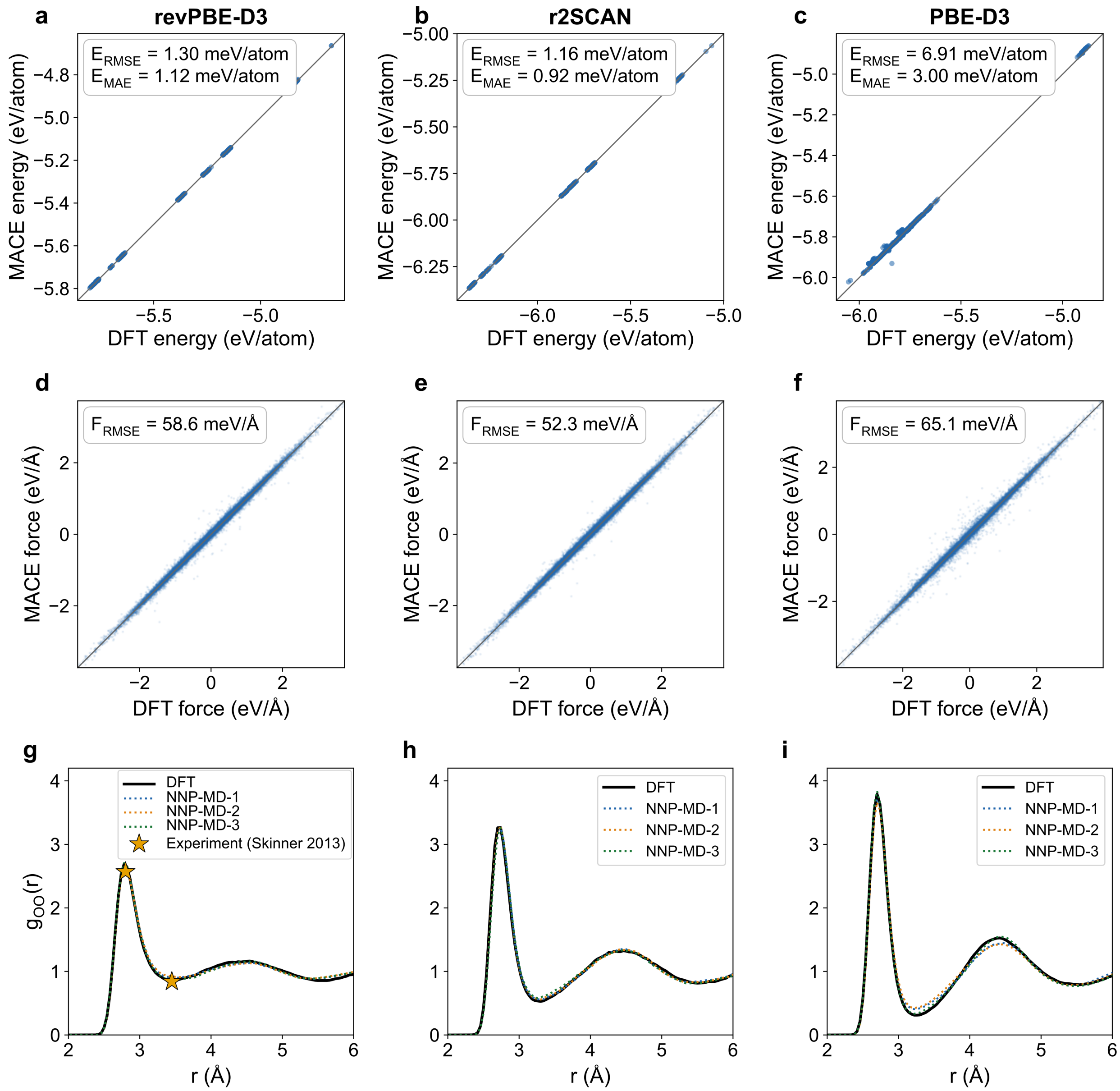

Figure S4: Multihead MACE model validation. Rows 1-2: Energy and force parity plots on validation sets (revPBE-D3, r2SCAN, PBE-D3 heads). Row 3: O-O RDF comparison of bulk water with VASP DFT and experiment,[35] showing recovery of water structure under improved functional choice. Bulk water MDs: 64 water molecules, 80 ps, cubic box 12.42 Å, 300 K NVT, 0.5 fs timestep; analysed over the equilibrated portion, the first ∼ 10 ps discarded

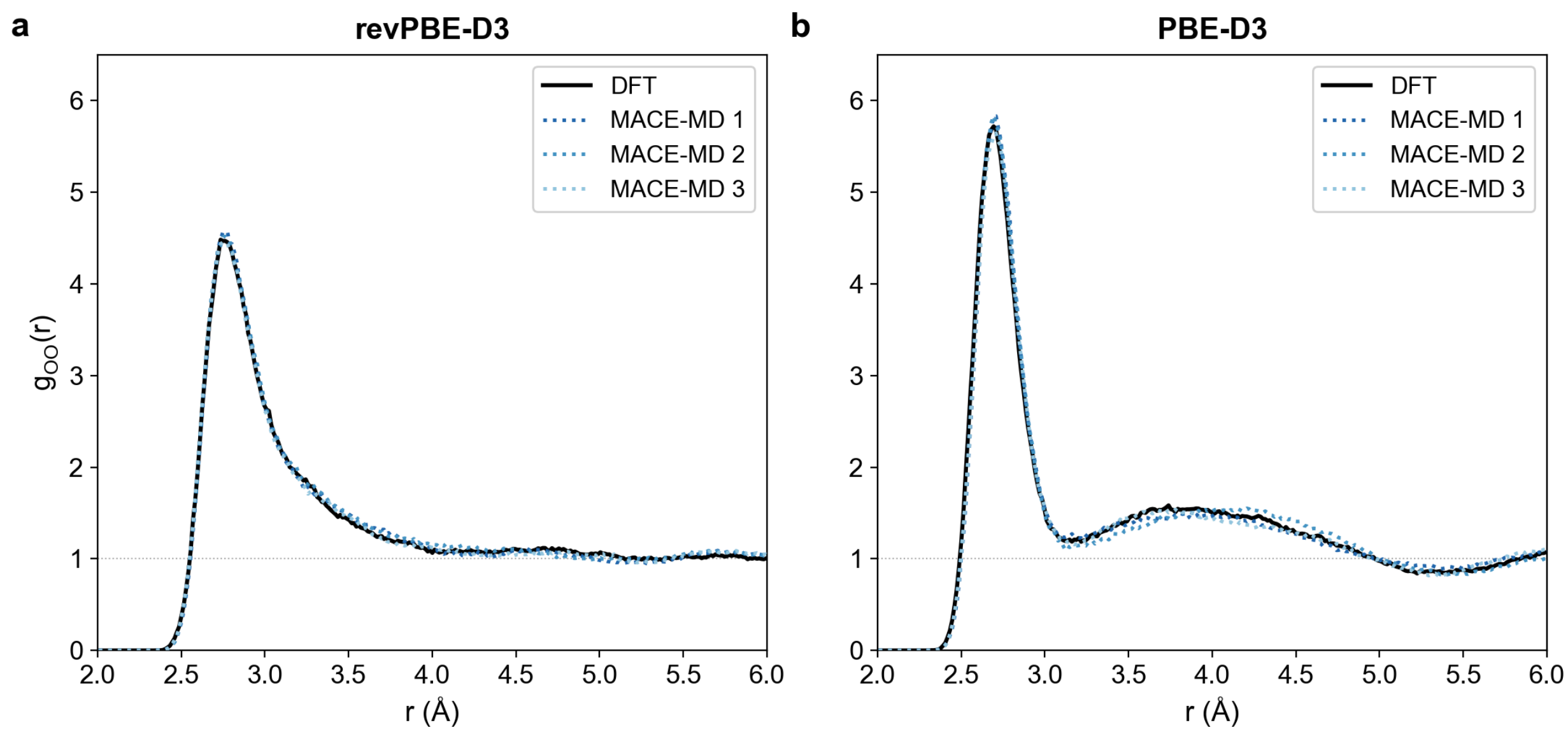

Figure S5: MACE-vs-DFT confined-water O–O radial distribution function benchmark for the 0.9Cl membrane pore. Water-oxygen $g_{OO}(r)$ from three independent MACE-MD replicas (blue dotted, MACE-MD 1–3) is compared against the reference DFT *ab initio* MD (solid black). (a) revPBE-D3 (b) PBE-D3. First-peak heights agree with the reference (revPBE-D3 +0.8%, t-statistic $= |t| = 0.56$; PBE-D3 +0.4%, $|t| = 0.24$) and peak positions to within 0.03 Å. DFT reference: a 50ps 300 K NVT (Nosé–Hoover) trajectory, 0.5 fs time-step, the first 10 ps discarded as equilibration.

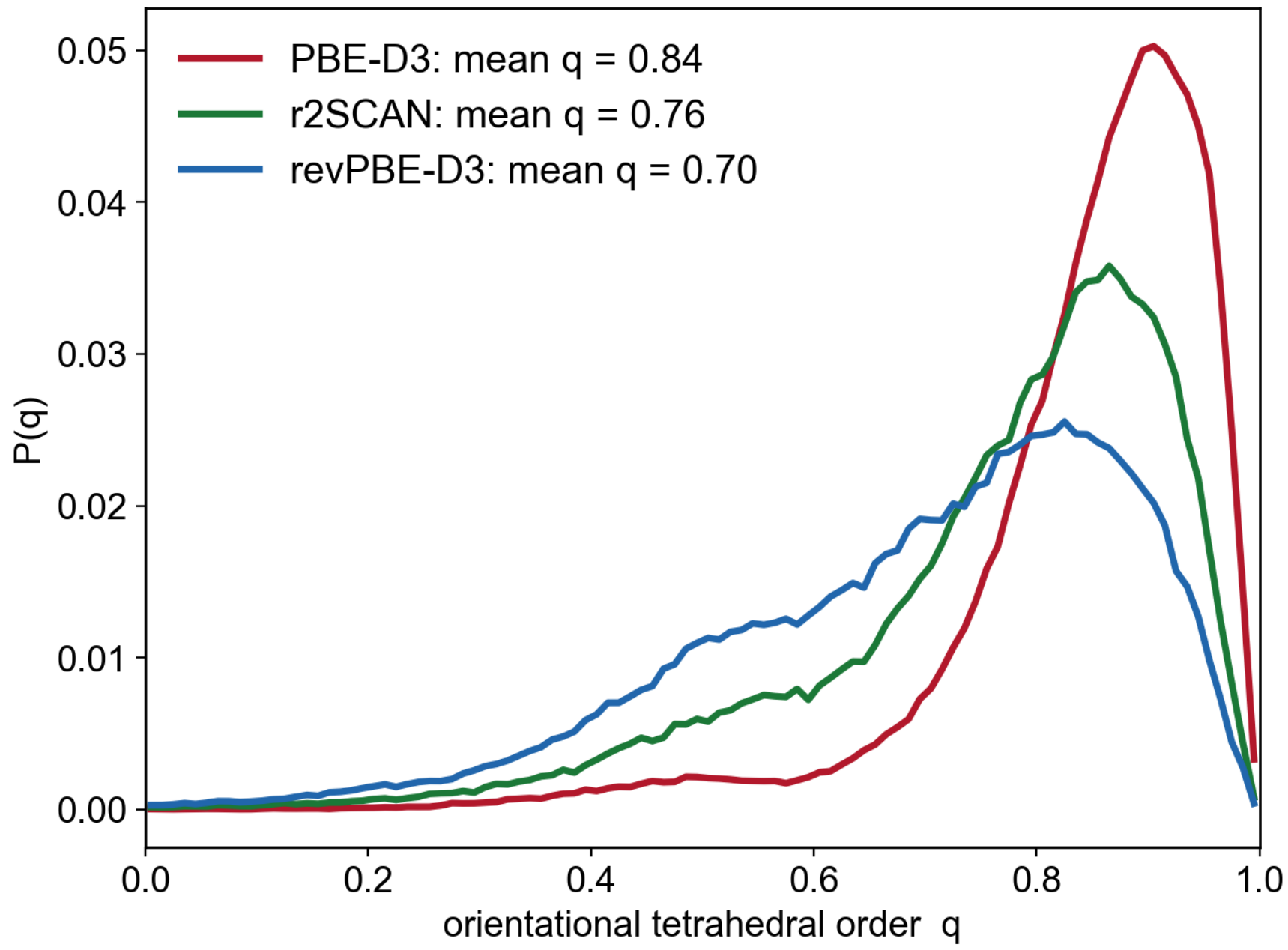

Figure S6: Orientational tetrahedral order q of bulk water from equilibrated *ab initio* molecular dynamics (64 water molecules, 80 ps, cubic box 12.42 Å, 300 K NVT, 0.5 fs timestep; analysed over the equilibrated portion, the first $\sim$ 10 ps discarded). q follows the Errington–Debenedetti definition over the four nearest water oxygens.[29,36] PBE-D3 over-structures (mean $q = 0.84$, unimodal at $q \approx 0.90$); revPBE-D3 (mean $q = 0.70$) reproduces the liquid-water value ($q \approx 0.67$, TIP4P/2005[32]); r2SCAN is intermediate (mean $q = 0.76$).

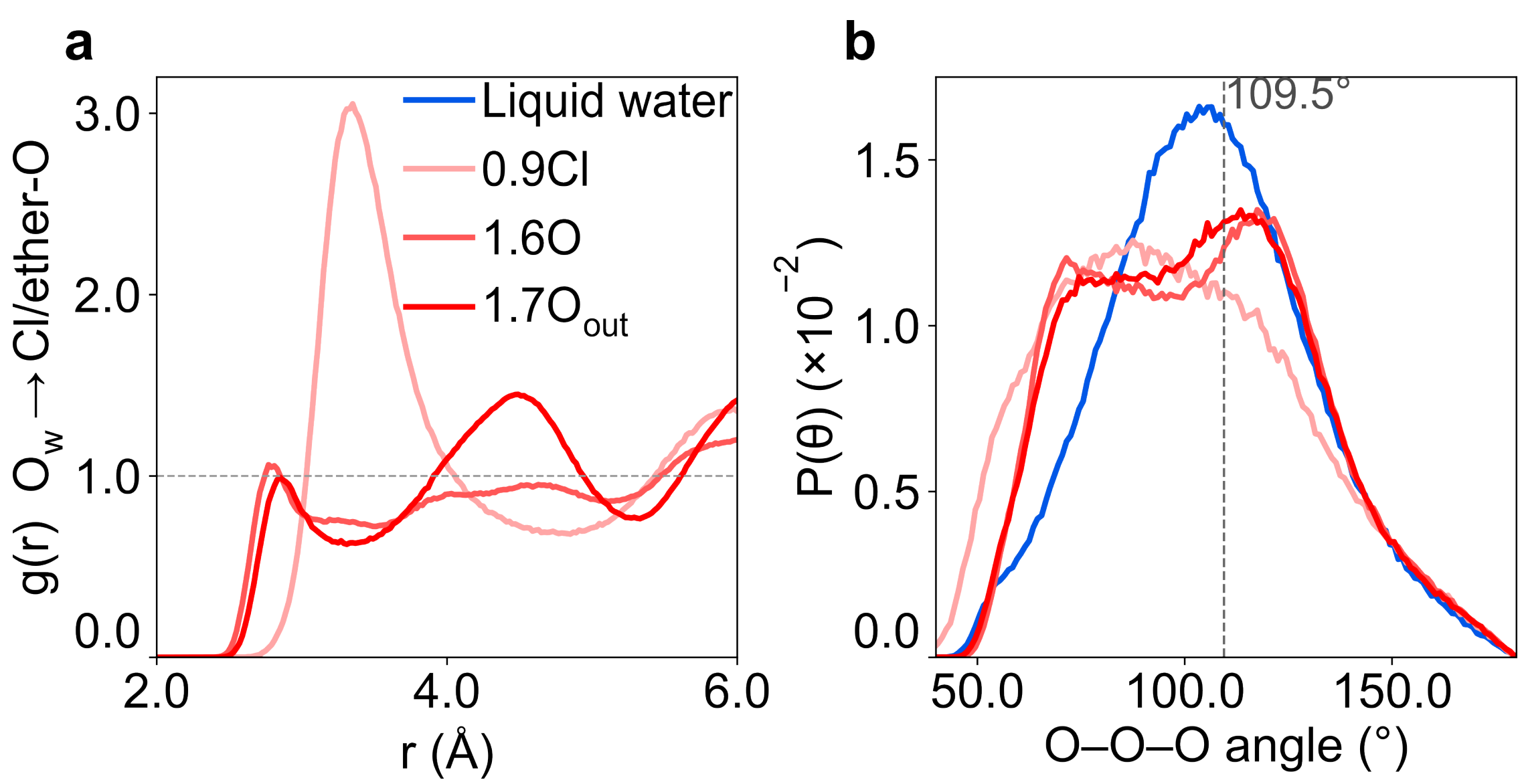

Figure S7: Confined water structure at revPBE-D3 (MACE), complementing Fig. ??: (a) radial distribution function between water oxygens and each pore's wall group (Cl for 0.9Cl, ether O for 1.6O and 1.7O_{out}); (b) O-O-O angle distribution of the water network, with the tetrahedral angle 109.5° marked.

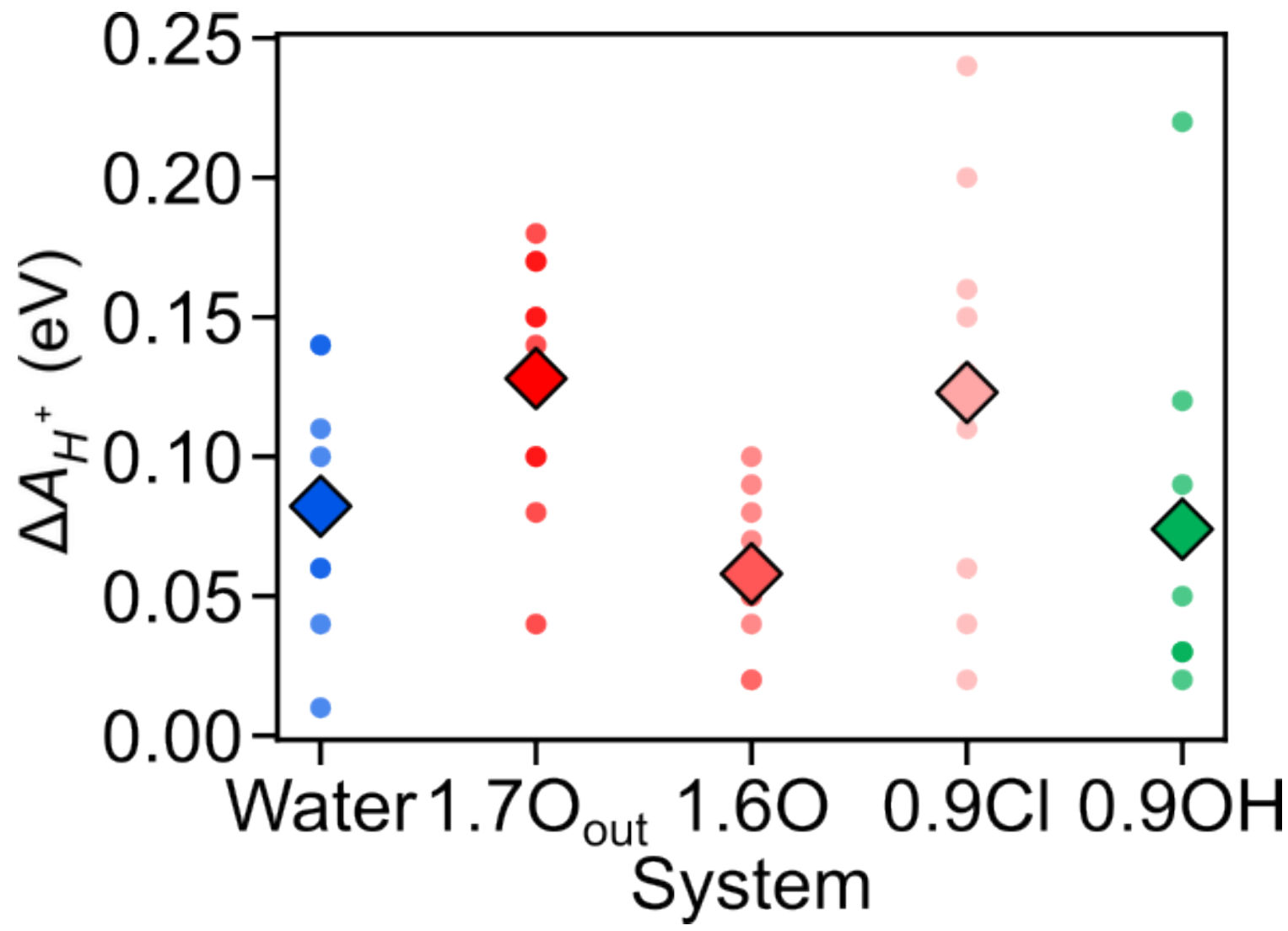

Figure S8: a) The proton transfer free-energy barriers (ΔA_{H^+}, eV) for all studied systems. The bulk-water free-energy barrier ($\Delta A_{H^+} \approx 0.08$ eV, $\approx 3\,k_BT$ at 300 K) is of the order of the thermal energy and consistent with the literature on aqueous proton transfer. Ab initio molecular dynamics of the excess proton give transfer barriers of order k_BT that grow with the donor–acceptor O–O distance (0.9–1.8 k_BT).[37] The experimental apparent activation energy for proton transfer in acidic water is 2.4 kcal mol^{-1}, $\approx 4\,k_BT$.[38]

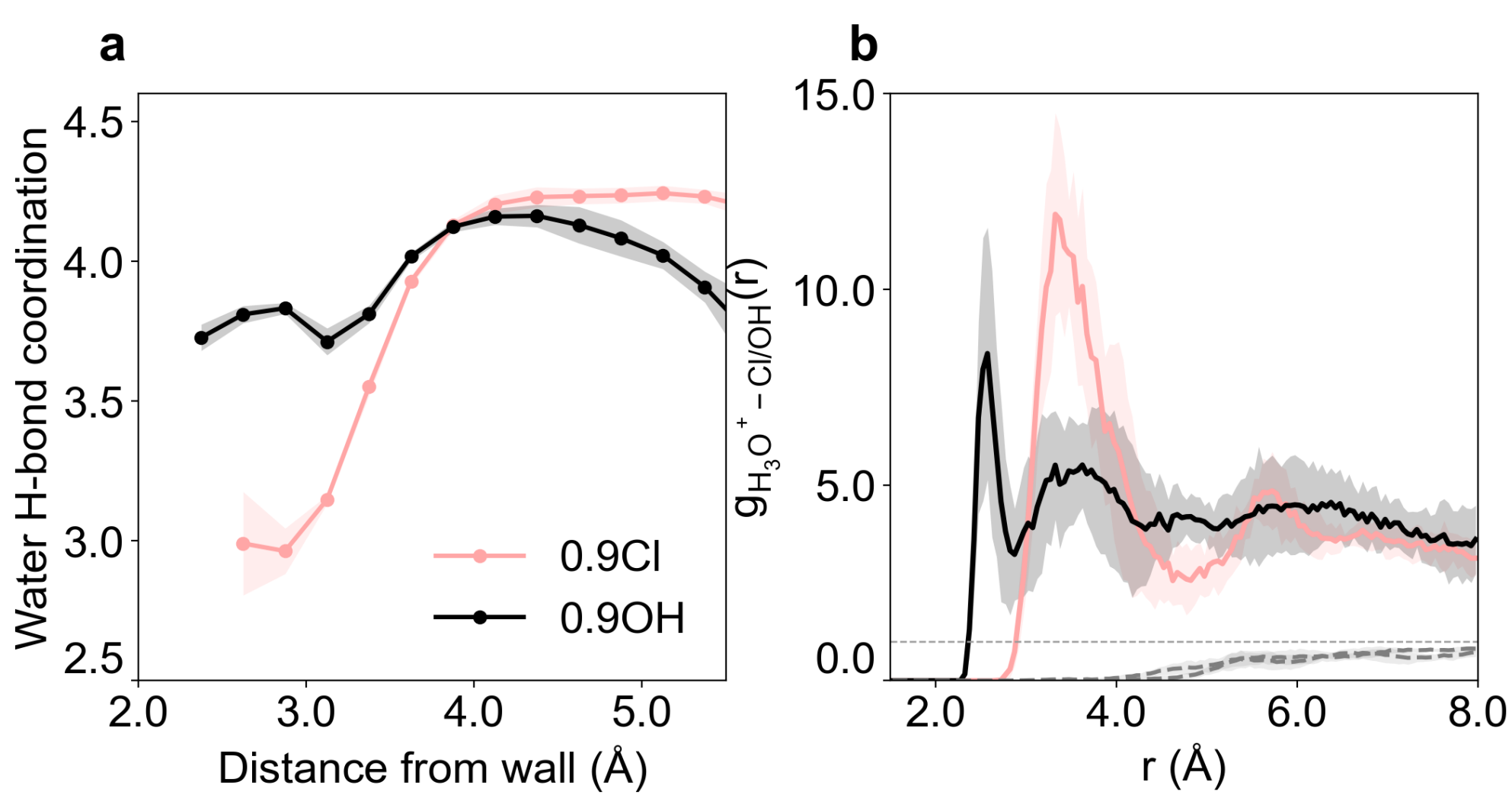

Figure S9: Wall coupling in 0.9Cl and the modified 0.9OH pore, computed at revPBE-D3. (a) Water hydrogen-bond coordination as a function of distance from the wall group; near the wall the OH pore preserves close to bulk coordination (about 3.8) whereas the Cl pore is depleted (about 3.1). (b) Radial distribution function between the excess proton (H_3O^+) and the wall group (Cl or OH); the proton contacts the hydroxyl oxygen (O*, the oxygen of the CH_2OH group in 0.9OH) at 2.5 Å, closer than the 3.4 Å chloride contact.

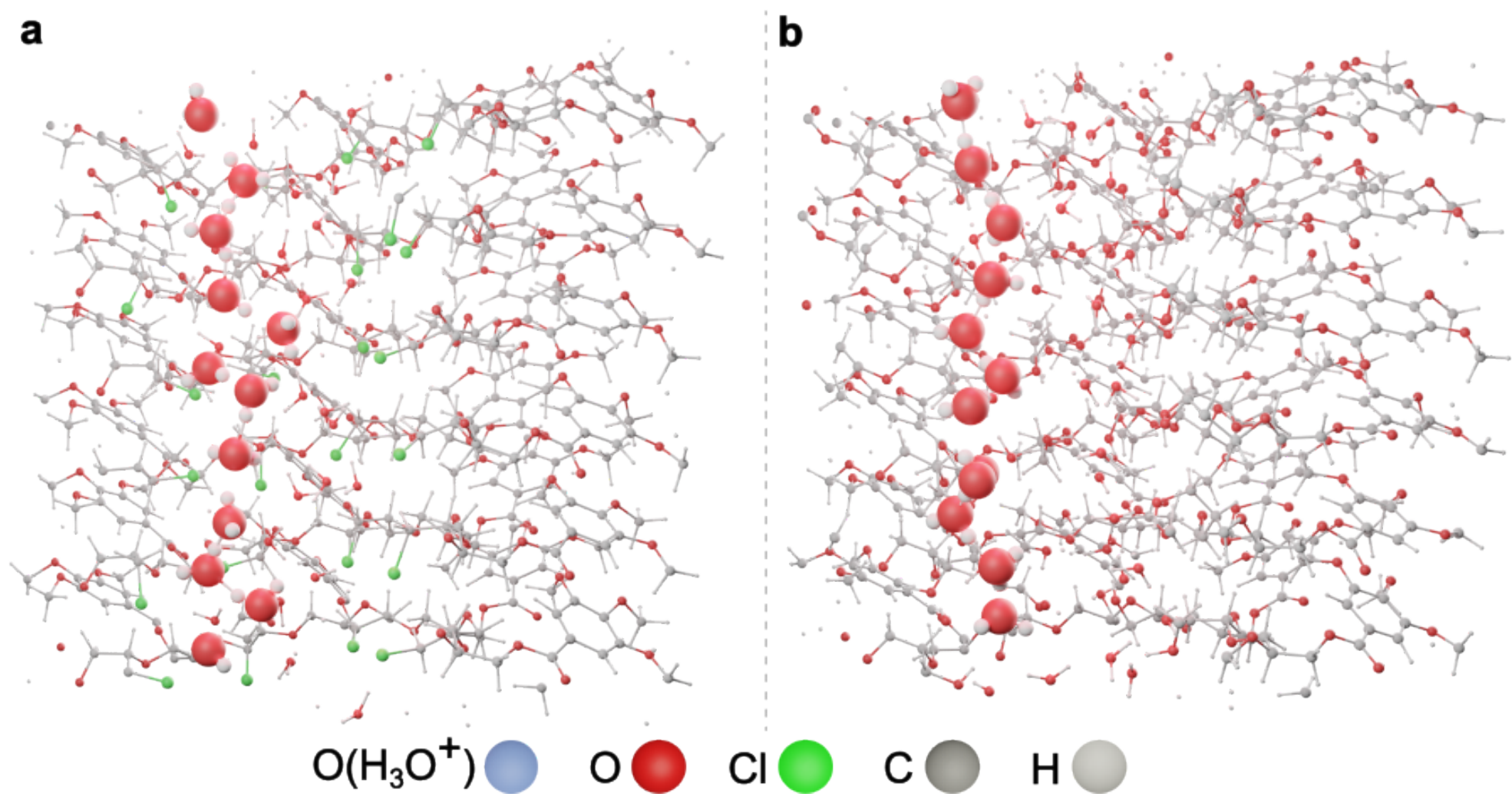

Figure S10: a) The illustrated cross-section of 0.9Cl (a) and 0.9OH (b) showing water wires.

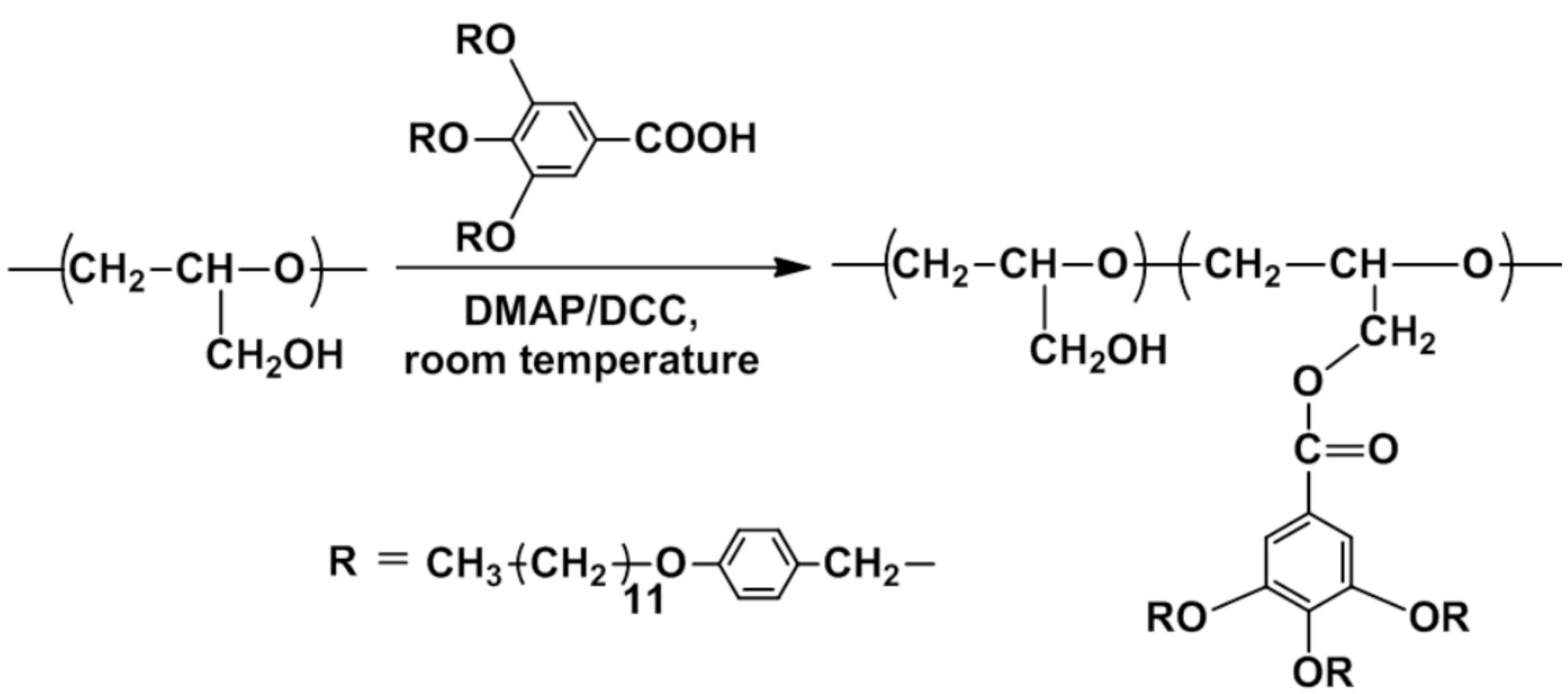

Figure S11: Chemical modification of linear polyglycidol (LPG).

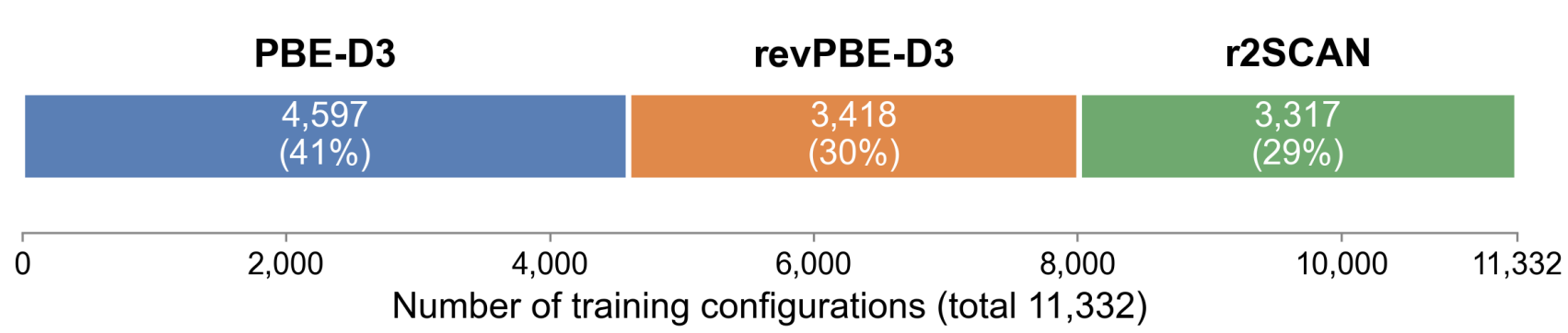

Figure S12: Composition of the multihead training set by DFT level: number of configurations labelled at each functional-specific head (PBE-D3, revPBE-D3, r2SCAN), 11,332 in total.

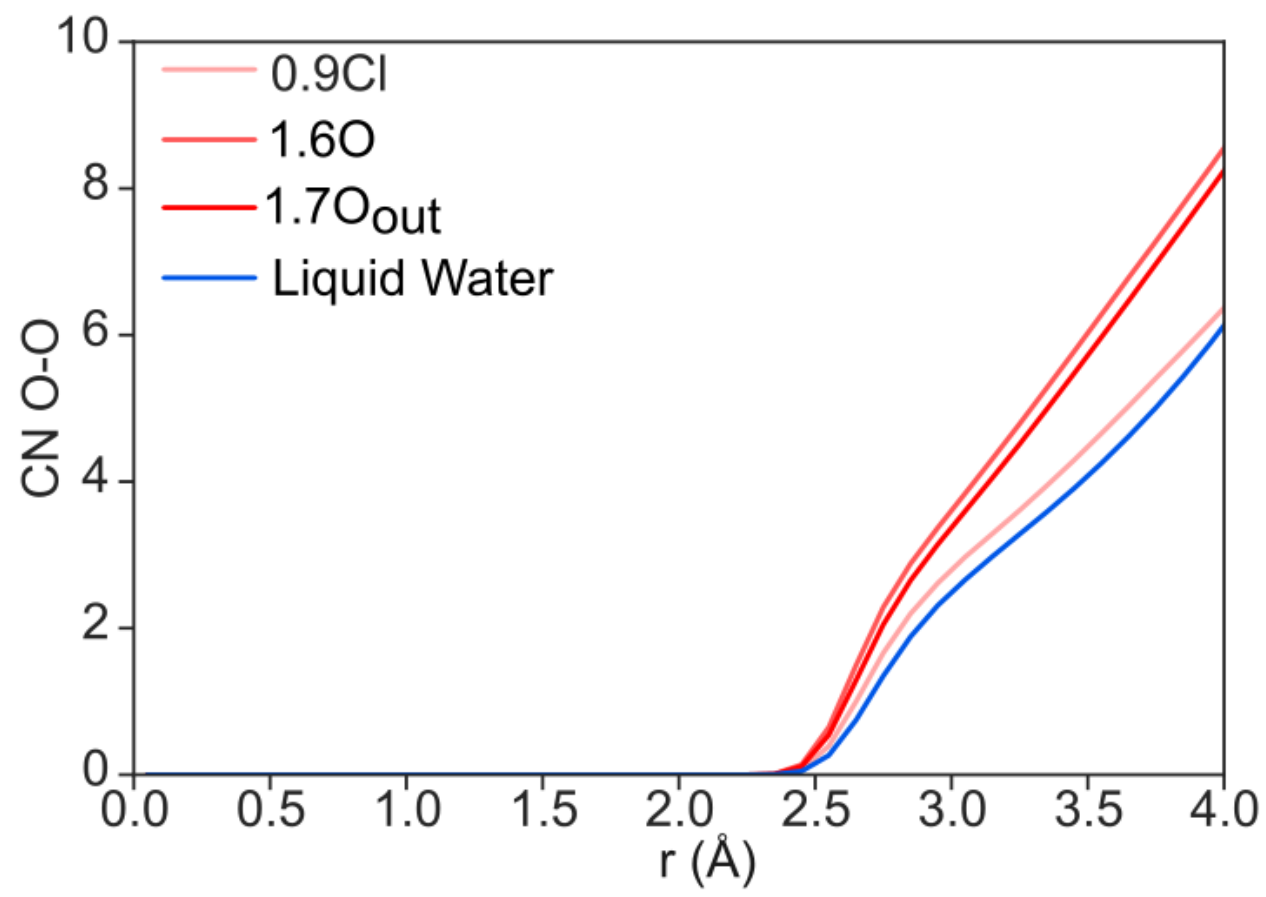

Figure S13: PBE-D3 (original submission). Coordination numbers of water oxygens computed from the O-O radial distribution function in the 0.9Cl, 1.6O and $1.7O_{out}$ pores and in liquid water. Compare Fig. S9a, computed at revPBE-D3.

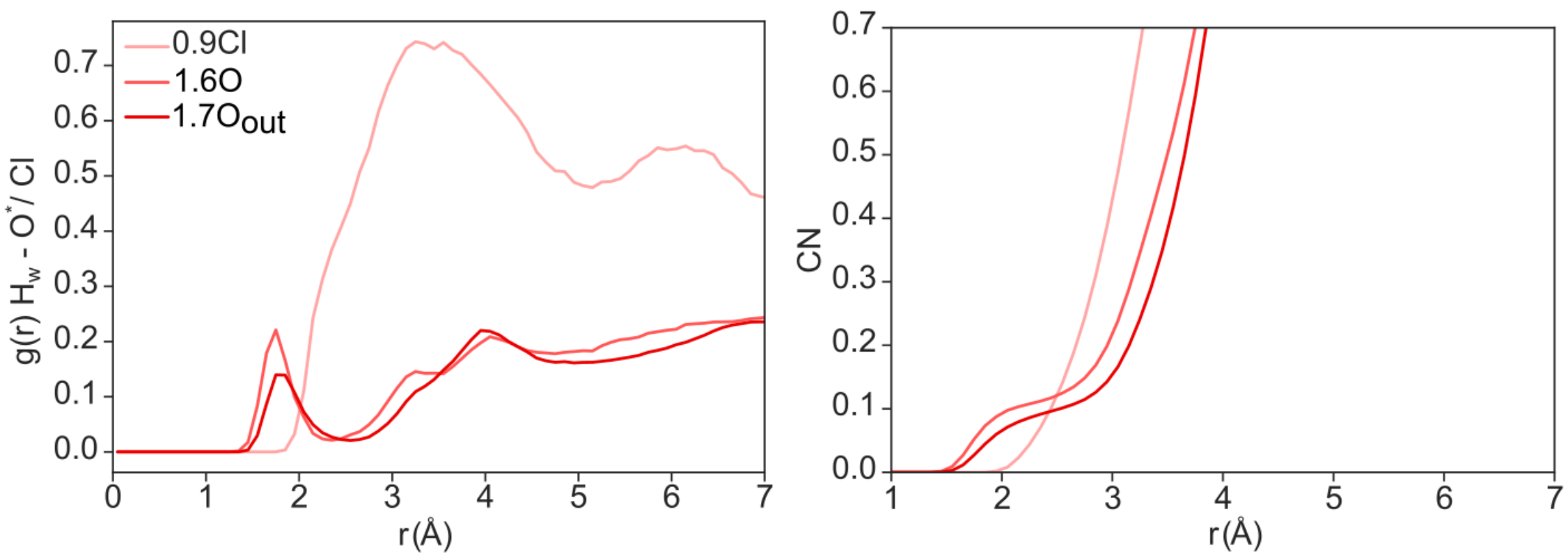

Figure S14: PBE-D3 (original submission). Radial distribution function between water hydrogens (H_w) and the pore wall groups, Cl in 0.9Cl and ether oxygens (O^*) in 1.6O and $1.7O_{out}$, with the corresponding coordination numbers on the right. Compare Fig. S9b, computed at revPBE-D3.

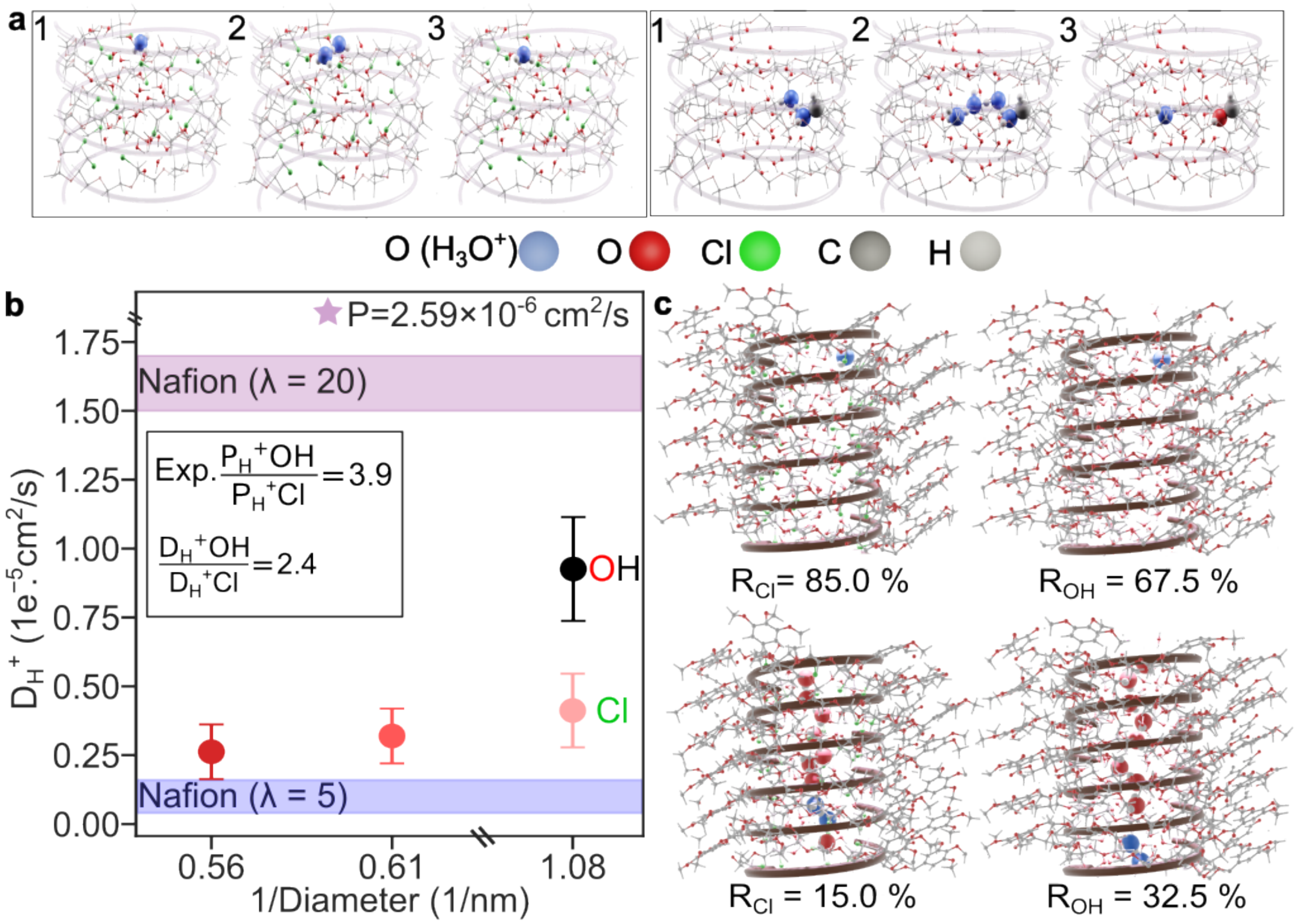

Figure S15: PBE-D3 (original submission), the figure this work replaces with Fig. ??. (a) Proton-transfer mechanism for 0.9Cl and 0.9OH. (b) Proton diffusion coefficients for all membranes, with the highest value in 0.9OH; at revPBE-D3 the highest values are found in the ether pores. Shaded lines mark Nafion at hydration levels $\lambda = 5$ and 20[39] and the star the measured Nafion 117 permeability. (c) Excess-proton residence near the pore wall against the central water for 0.9Cl and 0.9OH; the same contrast is obtained at revPBE-D3 and is larger there (Table. S6).

Supporting Tables

Table S1: The modification degree and yield obtained in the modification of PECH

Sample	PECH/RCOOK[a]	PECH (mol)	Solvent	Time (days)	Modification (%)	Yield (%)
PECH63	1:1.0	0.01	THF	8	63	89
PECH75	1:1.2	0.01	THF	8	75	85

[a] Stoichiometric amounts of TBAB used in each case.
[b] Average value determined by ^{1}H NMR.
[c] Calculated from average degree of modification.

Table S2: Structural, orientational, and columnar properties of water molecules and membranes across different systems. Mean orientational tetrahedral order (q) and distance between two disks in theoretical membrane model. The tetrahedral order q for Liquid Water, 1.6O, 1.7O$_{out}$ and 0.9Cl is computed with the revPBE-D3 head of the MACE model; the 0.9OH value is retained from the PBE-D3 trajectory.

System	q	Distance (Å)
Liquid Water	0.70	—
1.7O$_{out}$	0.50	4.47
1.6O	0.50	4.42
0.9Cl	0.37	4.16
0.9OH	0.66	4.19

In 0.9Cl the water oxygens approach the chloride closely (first peak near 3.3 Å) while the water hydrogens do not bond to it (cf. Fig. ??c), and the O-O-O angle distribution broadens away from the tetrahedral 109.5° in every pore, most strongly in 0.9Cl, mirroring the loss of tetrahedral order (Fig. S7).

Table S3: Experimental and theoretical water uptake values for different membrane models.

Membrane Model	H_2O/Ring	Water Uptake (%) (Theor./Exp.)
PECH75 (Exp.)	—	— / 7.5
LPG8 (Exp.)	—	— / 3.1
0.9Cl	13	3.8 / —
0.9OH	13	3.8 / —
1.6O	31	9.1 / —
1.7O$_{out}$	32	9.3 / —

Table S4: Permeability ($P \times 10^6$ cm^2/s), TAP values, and water uptake for different membranes.

**Membrane Model**	**TAP (%)**	$\boldsymbol{P}$ **×10⁶ (cm²/s)**	**Water Uptake (%)**
PECH63	63	0.56 ± 0.01	3.0 ± 1.3
PECH75	75	0.55 ± 0.01	3.3 ± 1.2
LPG8	11	2.2 ± 0.5	3.1 ± 1.2
LPG2	23	0.38 ± 0.01	–
LPG4	39	0.1 ± 0.03	–
Nafion 117	–	2.59 ± 0.05	32 ± 3.0

Table S5: The modification degree and yield for different LPG samples

**Sample**	**RCOOH (mmol)[a]**	**–OH/RCOOH**	**Time (days)**	**T (ºC)**	**Modification degree (%)[b]**	**Yield (%)**
LPG2	2	1:0.5	2	25	23	70
LPG4	2	1:1	2	25	39	81
LPG8	2	1:1	2	80	11	65

[a] Stoichiometric amounts of TBAB used in each case.
[b] Average value determined by ^{1}H NMR.

Table S6: Proton-transport quantities at the PBE-D3 level of the original submission and at the revPBE-D3 level of this work (Section). Uncertainties on the revPBE-D3 values are standard deviations over five independent 1 ns trajectories, four for 0.9Cl. The wall region is defined from the excess-proton position in this work and from the hydronium oxygen position in the original submission, so the residence percentages are close but not defined identically.

**Quantity**	**PBE-D3 (original)**	**revPBE-D3 (this work)**
Proton residence at wall, 0.9Cl	85%	94.4 ± 3.3%
Proton residence at wall, 0.9OH	67%	52.7 ± 4.7%
Mean wall dwell, 0.9Cl	not measured	3.8 ± 1.8 ps
Mean wall dwell, 0.9OH	not measured	0.33 ± 0.03 ps
$D_{H^+}(\text{OH})/D_{H^+}(\text{Cl})$	2.4	3.8
Measured $P_{H^+}(\text{OH})/P_{H^+}(\text{Cl})$	3.9	3.9

References

(1) Schmitz, C.; Keul, H.; Möller, M. Synthesis of Multi-Arm-Star Polyglycidols of Different Architecture Grafted with Polyacrylate Side Arms. *European Polymer Journal* **2009**, *45*, 2529–2539.

(2) Otera, J.; Nishikido, J. *Esterification: Methods, Reactions, and Applications, 2nd Edition*; Wiley, 2009.

(3) Bogdanowicz, K. A.; Bhosale, S. V.; Li, Y.; Vankelecom, I. F.; Garcia-Valls, R.; Reina, J. A.; Giamberini, M. Mimicking Nature: Biomimetic Ionic Channels. *Journal of Membrane Science* **2016**, *509*, 10–18.

(4) Tylkowski, B.; Castelao, N.; Giamberini, M.; Garcia-Valls, R.; Reina, J. A.; Gumí, T. The Importance of Orientation in Proton Transport of a Polymer Film Based on an Oriented Self-Organized Columnar Liquid-Crystalline Polyether. *Materials Science and Engineering: C* **2012**, *32*, 105–111.

(5) Montané, X.; Bhosale, S. V.; Reina, J. A.; Giamberini, M. Columnar Liquid Crystalline Polyglycidol Derivatives: A Novel Alternative for Proton-Conducting Membranes. *Polymer* **2015**, *66*, 100–109.

(6) Kresse, G.; Furthmüller, J. Efficiency of Ab-Initio Total Energy Calculations for Metals and Semiconductors Using a Plane-Wave Basis Set. *Computational Materials Science* **1996**, *6*, 15–50.

(7) Blöchl, P. E. Projector Augmented-Wave Method. *Physical Review B* **1994**, *50*, 17953–17979.

(8) Perdew, J. P.; Burke, K.; Ernzerhof, M. Generalized Gradient Approximation Made Simple. *Physical Review Letters* **1996**, *77*, 3865–3868.

(9) Zhang, Y.; Yang, W. Comment on "Generalized Gradient Approximation Made Simple". *Physical Review Letters* **1998**, *80*, 890–890.

(10) Grimme, S.; Ehrlich, S.; Goerigk, L. Effect of the Damping Function in Dispersion Corrected Density Functional Theory. *Journal of Computational Chemistry* **2011**, *32*, 1456–1465.

(11) Furness, J. W.; Kaplan, A. D.; Ning, J.; Perdew, J. P.; Sun, J. Accurate and Numerically Efficient r2SCAN Meta-Generalized Gradient Approximation. *The Journal of Physical Chemistry Letters* **2020**, *11*, 8208–8215.

(12) Nosé, S. A Unified Formulation of the Constant Temperature Molecular Dynamics Methods. *The Journal of Chemical Physics* **1984**, *81*, 511–519.

(13) Hoover, W. G. Canonical Dynamics: Equilibrium Phase-Space Distributions. *Physical Review A* **1985**, *31*, 1695–1697.

(14) Shuichi, N. Constant Temperature Molecular Dynamics Methods. *Progress of Theoretical Physics Supplement* **1991**, *103*, 1–46.

(15) Woo, T. K.; Margl, P. M.; Blöchl, P. E.; Ziegler, T. A Combined Car-Parrinello QM/MM Implementation for Ab Initio Molecular Dynamics Simulations of Extended Systems: Application to Transition Metal Catalysis. *The Journal of Physical Chemistry B* **1997**, *101*, 7877–7880.

(16) Sprik, M.; Ciccotti, G. Free Energy from Constrained Molecular Dynamics. *The Journal of Chemical Physics* **1998**, *109*, 7737–7744.

(17) Batatia, I.; Kovacs, D. P.; Simm, G.; Ortner, C.; Csanyi, G. MACE: Higher Order Equivariant Message Passing Neural Networks for Fast and Accurate Force Fields. NeurIPS 2022 Conference. 2022; pp 11423–11436.

(18) Reddi, S. J.; Kale, S.; Kumar, S. On the Convergence of Adam and Beyond. International Conference on Learning Representations (ICLR). 2018.

(19) Izmailov, P.; Podoprikhin, D.; Garipov, T.; Vetrov, D.; Wilson, A. G. Averaging Weights Leads to Wider Optima and Better Generalization. Proceedings of the Thirty-Fourth Conference on Uncertainty in Artificial Intelligence (UAI). 2018; pp 876–885.

(20) Schneider, L.; Wistuba, M.; Klein, A.; Golebiowski, J.; Zappella, G.; Merra, F. A. Hyperband-Based Bayesian Optimization for Black-Box Prompt Selection. Proceedings of the 42nd International Conference on Machine Learning (ICML). 2025.

(21) Seung, H. S.; Opper, M.; Sompolinsky, H. Query by Committee. Proceedings of the Fifth Annual Workshop on Computational Learning Theory. 1992; pp 287–294.

(22) Martínez, J. M.; Martínez, L. Packing Optimization for Automated Generation of Complex System's Initial Configurations for Molecular Dynamics and Docking. *Journal of Computational Chemistry* **2003**, *24*, 819–825.

(23) Martínez, L.; Andrade, R.; Birgin, E. G.; Martínez, J. M. PACKMOL: A package for building initial configurations for molecular dynamics simulations. *Journal of Computational Chemistry* **2009**, *30*, 2157–2164.

(24) Montero de Hijes, P.; Dellago, C.; Jinnouchi, R.; Schmiedmayer, B.; Kresse, G. Comparing Machine Learning Potentials for Water: Kernel-based Regression and Behler-Parrinello Neural Networks. *The Journal of Chemical Physics* **2024**, *160*, 114107.

(25) Schran, C.; Brezina, K.; Marsalek, O. Committee Neural Network Potentials Control Generalization Errors and Enable Active Learning. *The Journal of Chemical Physics* **2020**, *153*, 104105.

(26) Gomes-Filho, M. S.; Torres, A.; Rocha, A. R.; Pedroza, L. S. Size and Quality of

Quantum Mechanical Data Set for Training Neural Network Force Fields for Liquid Water. *The Journal of Physical Chemistry B* **2023**, *127*, 1422–1428.

(27) Cheng, B.; Engel, E. A.; Behler, J.; Dellago, C.; Ceriotti, M. Ab Initio Thermodynamics of Liquid and Solid Water. *Proceedings of the National Academy of Sciences* **2019**, *116*, 1110–1115.

(28) Morawietz, T.; Singraber, A.; Dellago, C.; Behler, J. How van Der Waals Interactions Determine the Unique Properties of Water. *Proceedings of the National Academy of Sciences* **2016**, *113*, 8368–8373.

(29) Chau, P.-L.; Hardwick, A. J. A New Order Parameter for Tetrahedral Configurations. *Molecular Physics* **1998**, *93*, 511–518.

(30) Zhao, G.; Yan, J. L.; Yu, Y. J.; Ding, M. C.; Zhao, X. G.; Wang, H. Y. Relationship between Structural Order and Water-like Anomalies in Metastable Liquid Silicon: Ab Initio Molecular Dynamics. *Scientific Reports* **2017**, *7*, 39952.

(31) Chen, M.; Ko, H.-Y.; Remsing, R. C.; Calegari Andrade, M. F.; Santra, B.; Sun, Z.; Selloni, A.; Car, R.; Klein, M. L.; Perdew, J. P.; Wu, X. Ab Initio Theory and Modeling of Water. *Proceedings of the National Academy of Sciences* **2017**, *114*, 10846–10851.

(32) Duboué-Dijon, E.; Laage, D. Characterization of the Local Structure in Liquid Water by Various Order Parameters. *The Journal of Physical Chemistry B* **2015**, *119*, 8406–8418.

(33) Maginn, E. J.; Messerly, R. A.; Carlson, D. J.; Roe, D. R.; Elliot, J. R. Best Practices for Computing Transport Properties 1. Self-Diffusivity and Viscosity from Equilibrium Molecular Dynamics. *Living Journal of Computational Molecular Science* **2019**, *1*, 6324.

(34) Yeh, I.-C.; Hummer, G. System-Size Dependence of Diffusion Coefficients and Viscosities from Molecular Dynamics Simulations with Periodic Boundary Conditions. *The Journal of Physical Chemistry B* **2004**, *108*, 15873–15879.

(35) Skinner, L. B.; Huang, C.; Schlesinger, D.; Pettersson, L. G. M.; Nilsson, A.; Benmore, C. J. Benchmark Oxygen-Oxygen Pair-Distribution Function of Ambient Water from x-Ray Diffraction Measurements with a Wide Q-Range. *The Journal of Chemical Physics* **2013**, *138*, 074506.

(36) Errington, J. R.; Debenedetti, P. G. Relationship between Structural Order and the Anomalies of Liquid Water. *Nature* **2001**, *409*, 318–321.

(37) Brünig, F. N.; Rammler, M.; Adams, E. M.; Havenith, M.; Netz, R. R. Spectral Signatures of Excess-Proton Waiting and Transfer-Path Dynamics in Aqueous Hydrochloric Acid Solutions. *Nature Communications* **2022**, *13*, 4210.

(38) Carpenter, W. B.; Lewis, N. H. C.; Fournier, J. A.; Tokmakoff, A. Entropic Barriers in the Kinetics of Aqueous Proton Transfer. *The Journal of Chemical Physics* **2019**, *151*, 034501.

(39) Cui, R.; Li, S.; Yu, C.; Zhou, Y. The Evolution of Hydrogen Bond Network in Nafion via Molecular Dynamics Simulation. *Macromolecules* **2023**, *56*, 1688–1703.
